# Effectiveness of self‐financing patient‐led support groups in the management of hypertension and diabetes in low‐ and middle‐income countries: Systematic review

**DOI:** 10.1111/tmi.13842

**Published:** 2022-12-23

**Authors:** Richard E. Sanya, Erin Stewart Johnston, Peter Kibe, Mahmoud Werfalli, Sloan Mahone, Naomi S. Levitt, Kerstin Klipstein‐Grobusch, Gershim Asiki

**Affiliations:** ^1^ Chronic Diseases Management Unit African Population and Health Research Center Nairobi Kenya; ^2^ Julius Global Health, Julius Center for Health Sciences and Primary Care University Medical Center Utrecht, Utrecht University Utrecht The Netherlands; ^3^ Department of Family and Community Medicine, Faculty of Medicine University of Benghazi Benghazi Libya; ^4^ Oxford Centre for the History of Science, Medicine and Technology Oxford University Oxford UK; ^5^ Chronic Disease Initiative for Africa Department of Medicine, Faculty of Health Science University of Cape Town Cape Town South Africa; ^6^ Division of Epidemiology and Biostatistics, School of Public Health, Faculty of Health Sciences University of the Witwatersrand Johannesburg South Africa

**Keywords:** chronic care, diabetes, financing, hypertension, low‐ and middle‐income countries, patient support groups, systematic review

## Abstract

**Objective:**

There is insufficient evidence on the role of self‐financing patient support groups in the control of blood pressure (BP) and/or diabetes in low‐ and middle‐income countries (LMICs). We conducted a systematic review to investigate the effectiveness of these groups in BP and glycaemic control.

**Methods:**

We searched PubMed, Embase, SCOPUS, Web of Science, Global Health, African Journals Online, CINAHL and African Index Medicus for published peer‐reviewed articles from inception up to November 2021. Grey literature was obtained from OpenGrey. Studies on patient support groups for hypertension and/or diabetes with a component of pooling financial resources, conducted in LMICs, were included. Narrative reviews, commentaries, editorials and articles published in languages other than English and French were excluded. Study quality and risk of bias were assessed using the National Institutes of Health Quality assessment tool and the revised Cochrane risk‐of‐bias tool. Results are reported according to PRISMA guidelines.

**Results:**

Of 724 records screened, three studies met the criteria: two trials conducted in Kenya and a retrospective cohort study conducted in Cambodia. All studies reported improvement in BP control after 12 months follow‐up with reductions in systolic BP of 23, 14.8, and 16.9 mmHg, respectively. Two studies reported diabetes parameters. The first reported improvement in HbA1c (reduction from baseline 10.8%, to 10.6% at 6 months) and random blood sugar (baseline 8.9 mmol/L, to 8.5 mmol/L at 6 months) but these changes did not achieve statistical significance. The second reported a reduction in fasting blood glucose (baseline—216 mg/dl, 12 months—159 mg/dl) in diabetic patients on medication.

**Conclusion:**

Self‐financing patient support groups for diabetes and hypertension are potentially effective in the control of BP and diabetes in LMICs. More studies are needed to add to the scarce evidence base on the role of self‐financing patient support groups.

## INTRODUCTION

WHO estimates that 1.13 billion individuals have hypertension worldwide: two‐thirds of these are living in low and middle‐income countries (LMICs) [1]. In 2019, the International Diabetes Federation (IDF) estimated that 463 million individuals had diabetes worldwide and 19.4 million were living in sub‐Saharan Africa [2]. The burden of these chronic conditions is rising with LMICs disproportionately affected [2, 3, 4], posing a great challenge to financing of care by households and healthcare systems [5]. Many LMICs are failing to cope with the current burden of these chronic diseases [6].

Patient support groups are a potential self‐management strategy that can improve the care for chronic diseases such as hypertension and diabetes. Patient‐led support groups represent an ideological shift away from the perception of patients as ‘passive’ recipients of treatment to empowered individuals who are partners in the effective management of their own health [7]. Peer support has been shown to improve healthy behaviours among patients with diabetes in under‐resourced countries [8, 9]. Patient self‐support groups have been recognised by WHO in HIV care and were included in the HIV treatment guidelines of 2013 [10]. The roles of the support groups range from improving treatment adherence, providing emotional support, inducing behavioural changes and adjustments to lifestyle, self‐monitoring and reporting of disease [9, 11]. Thus, these groups have generated substantial interest as an approach for reaching low‐income patients.

Considering the scarcity of public resources including financing of care and human resources in LMICs for managing patients with hypertension and diabetes, innovative self‐sustaining approaches such as peer‐led patient support groups are needed to improve the care of people with chronic diseases. Essential medicines needed for treating these diseases are not readily available in the public sector in many LMICs [12, 13]. In many LMICs, health insurance coverage is low, making self‐financing patient support groups an alternative approach for low‐income patients who are mostly uninsured to access medicines at a subsidised cost [14].

There is paucity of evidence on the presence and effectiveness of self‐financing patient support groups for hypertension and diabetes in LMICs. We conducted this systematic review to assess the effectiveness of self‐financing patient support groups in LMICs with regard to blood pressure and glycaemic control.

## METHODS

### Review registration and reporting

The protocol for the systematic review was prospectively registered in the International Prospective Register for Systematic Reviews (PROSPERO; CRD42021282203) [15] and the findings reported according to the Preferred Reporting Items for Systematic Reviews (PRISMA) [16] (File S1).

### Eligibility criteria

Self‐financing patient support groups were defined as self‐help, peer‐support groups of patients set up by patients that pool group financial resources to support the purchase of medicines and/or self‐care equipment. We included studies (with no restriction to study design) involving patient support groups for either diabetes or hypertension or both, that had at least an element of pooling group financial resources and were conducted in LMICs as defined by the World Bank [17]. Narrative reviews, commentaries, editorials, non‐original articles and articles published in languages other than English and French were excluded.

### Information sources and search strategy

The electronic bibliographic databases MEDLINE (via PubMed), Embase, Scopus, Web of Science, Global Health, African Journals Online, Cumulative Index to Nursing and Allied Health Literature (CINAHL) and African Index Medicus (AIM) were searched to identify relevant articles. Additionally, grey literature was searched from OpenGrey [18]. The literature search was conducted using the following keywords: (‘hypertension’[MeSH] OR hypertension[tiab] OR hypertensive[tiab] OR ‘high blood pressure’[tiab] OR ‘blood pressure’ OR diabetes[tiab] OR diabet*[tiab] OR dm2[tiab] OR niddm[tiab] OR dm 2[tiab] OR t2d[tiab] OR ‘dm type 2’[tiab] OR ‘dm type II’[tiab] OR dm1[tiab] OR iddm[tiab] OR dm 1[tiab] OR t1d[tiab] OR ‘dm type 1’[tiab] OR ‘dm type I’[tiab] OR ‘Diabetes Mellitus’[Mesh]) AND (‘support group*’[tiab] OR buddy[tiab] OR ‘self‐help group*’ [tiab] OR ‘peer group*’ [tiab] OR ‘informal group*’[tiab] OR ‘social group’[tiab] OR ‘Volunteers’[Mesh] OR ‘Self‐help groups’[Mesh] OR ‘Peer group’[Mesh] OR ‘peer/microfinance’[tiab] OR ‘group medical visits’[tiab]) AND (financ*[tiab] OR cash[tiab] OR fund[tiab] OR contribution[tiab] OR support[tiab] OR backing[tiab] OR aid[tiab] OR microfinance[tiab]). The search strategy for PubMed was adapted to match the syntax and subject headings for the other databases. The full search matrix is provided as File S2.

### Selection process

Articles from the various databases were retrieved and exported to *Endnote X.9.3.3* software where duplicates were sorted and removed. Then, the articles were uploaded to Rayyan [19], a web‐based software program that allows multiple reviewers to screen and select articles for review. Titles and abstracts were independently screened by three reviewers (RES, ESJ and PK) and a fourth reviewer (KK‐G) was involved as a tiebreaker to resolve any disagreements. Full texts of the eligible article were obtained for further screening.

### Data collection process

Data were extracted from the eligible studies using the selection criteria to spreadsheets designed for screening and selection of articles. A standardised data extraction form was adapted to extract data (File S3). Data extracted included citation, design, study year, year of publication, key findings and the quality of evidence.

### Data items

For each study, data were extracted on study characteristics (citation, design, study year and year of publication), key findings and the quality of evidence. For the latter, internal and external validity was assessed, and the quality of evidence was rated as strong, medium or weak. The primary outcome of the review was the impact of self‐financing patient support groups on blood pressure and glycaemic control.

The ideal outcome indicators for hypertension are changes in systolic and diastolic blood pressure. For diabetes, change in glycated haemoglobin would be the ideal measure because it measures control of blood sugar over a 3‐month period. Where HbA1c is not reported, blood glucose (random or fasting) was considered.

### Assessment of quality and risk of bias

An independent critical appraisal of retrieved articles was performed (by ESJ and MW) using standardised critical appraisal checklists. The Revised Cochrane risk‐of‐bias tool for cluster‐randomised trials (RoB 2 CRT, version 18 March 2021) was used to assess the risk of bias for cluster‐randomised trials. For cross‐sectional, cohort studies, case studies and case series the National Institutes of Health Quality assessment tool for observational, cohort and cross‐sectional studies were used [20].

### Synthesis methods

A narrative synthesis of the findings, documenting all the key outcome measures from the studies included in this review, is provided. Due to the variability in study designs, a meta‐analysis was not undertaken, and study heterogeneity and sensitivity analyses were not conducted. The results are summarised from all studies that report outcomes. The impact of self‐financing patient support groups was based on the magnitude of effect reported in individual studies, the quality of the body of evidence and consistency of results across the studies.

### Patient and public involvement

Patients or the public were not involved in the design, or conduct, or reporting, or dissemination plans of our systematic review.

## RESULTS

### Study selection

**FIGURE 1:**
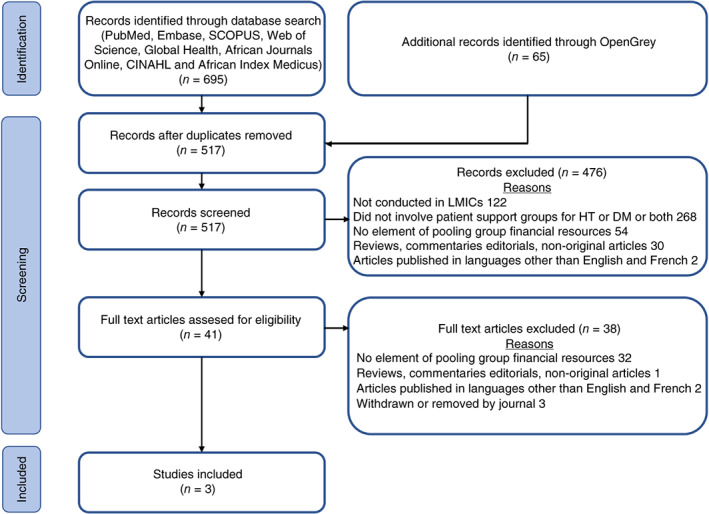
Preferred reporting items for systematic reviews and meta‐analyses (PRISMA) flow diagram. DM, diabetes mellitus; HT, hypertension; LMICs, low and middle income countries

As the search and selection process in Figure 1 shows, the search yielded 724 articles. Of these, 207 duplicates were removed, and titles and abstracts of 517 articles were screened. Forty‐one articles were retained for full‐text screening and three articles met the inclusion criteria and were included in the systematic review. These articles, published between 2017 and 2021, showcase two studies conducted in Kenya and one in Cambodia.

### Study characteristics

The selected studies are described in detail in Table 1. All were conducted in predominantly rural areas (Sinoko for Reference [21]) in Kenya, Kisumu, Uasin Gishu, Busia and Kisumu counties in Kenya [22] and Takeo province in Cambodia [23]. These studies include a randomised controlled trial [22], a before‐after study [21] and a retrospective cohort study [23]. All studies investigated self‐financing patient support groups and focussed on patients with or at risk of diabetes, and patients with hypertension. A total of 5999 participants were included across all the studies and data collection took place between 2007 and 2018.

**TABLE 1:** Description of studies included in the systematic review of self‐financing patient support groups in the management of hypertension and diabetes in low‐ and middle‐income countries

Study title	Country	Study design	Outcomes	Time points	No. of participants	Population	Intervention	Control/comparator	Financing
Impact of bridging income generation with group integrated care (BIGPIC) on hypertension and diabetes in rural Western Kenya. [21]	Kenya	Before‐After	Blood pressure Random blood glucose Glycated haemoglobin	Baseline 3 months 6 months 9 months 12 months	879	Patients with hypertension and/or diabetes	Screen for hypertension and diabetes in the community Link positively screened patients to peer/microfinance groups Integrate health education and agri‐business advice into group meetings Deliver care (consultations, point of care laboratory tests and medications) directly at group meetings Enhance economic sustainability through the availability of loans and pursuing income‐generating activities Use incentives delivered through group competitions to generate demand for care	Historical control group (community screening done and patients referred to government health facilities for care)	Microfinance (groups pool finances through member contributions)
Group medical visit and microfinance intervention for patients with diabetes or hypertension in Kenya. [22]	Kenya	Four‐arm cluster‐randomised trial	Blood pressure 10‐year cardiovascular disease event risk (assessed using the QRISK3 score Total cholesterol LDL‐cholesterol International wealth index Livestock ownership	Baseline 3 months 12 months	2890	≥35 years old Patients with diabetes Individuals with impaired fasting glucose Individuals with increased risk of diabetes (as assessed using the Leicester Risk Assessment Score) Patients with hypertension	Usual (multi‐component clinical) care plus microfinance Group medical visits Group medical visits with microfinance	Usual (multi‐component clinical) care	Microfinance (groups pool finances through member contributions)
Evaluation of a multi‐faceted diabetes care program including community‐based peer educators in Takeo province, Cambodia‐ 2007–2013. [23]	Cambodia	Retrospective cohort	Fasting blood sugar Blood pressure	Baseline 6 months 12 months 18 months 24 months	2230	Individuals with fasting blood glucose >126 mg/dl or postprandial blood glucose >180 mg/dL	Peer educators (individuals with diabetes trained in diabetes and hypertension care) conducted group meetings for diabetes self‐management education Peer educators monitored patients' fasting blood glucose and blood pressure checks A revolving drug fund was set up by the study and participants had access to buy prescribed medication	None	Revolving drug fund

Abbreviations: LDL‐cholesterol, Low‐density lipoprotein cholesterol; QRISK3, Cardiovascular risk score 3.

The studies in Kenya [21] and [22] were part of an integrated care programme that sought to identify patients in the communities, link them to create microfinance groups, provide education and health care within the groups and improve economic sustainability through member contributions, availability of loans and involvement in other income generation activities especially agriculture. The patients formed microfinance groups to mobilise and manage their own savings. The funds were used to pay for medical consultations, laboratory tests and medicines. In addition, the funds were made available to members as affordable loans. The interest generated was ploughed back into the groups. The before‐and‐after study by Pastakia et al. [21] investigated the impact of the care programme over 12 months with other time points at 3, 6 and 9 months. Vedanthan et al. [22], in a four‐arm cluster‐randomised trial design, investigated the impact of group medical visits (GMV) and microfinance on blood pressure, 10‐year cardiovascular disease event risk (assessed using the QRISK3 score), total cholesterol, LDL‐cholesterol, international wealth index and livestock ownership over 12 months.

For the Cambodia model described in Taniguchi et al.'s Reference [23], a non‐governmental organisation (NGO) provided care for hypertension and diabetes through peer support groups. Key to this was the setting up of a revolving drug fund, where medicines are bought in bulk and stocked at pharmacies and members pay for them at subsidised prices. The fund was set up by the NGO. The revenue generated was used to sustain the supply of medicines. The study evaluated the impact of the programme on fasting blood glucose and blood pressure over 24 months.

### Study quality and risk of bias assessment

The results from the National Institutes of Health Quality assessment tool are shown in File S2. All studies were of fair quality. In Vedanthan et al. [22], considering the nature of the intervention, the study participants and providers were not blinded to the group assignment. Secondly, the outcome assessors were not blinded to the participant group assignments. Taniguchi and Pastakia [21] did not document blinding of the outcome assessors. There was no justification and no power calculation for the sample size used in the studies Taniguchi et al. [23] and Pastakia et al. [21]. The risk of bias was assessed for Vedanthan et al. [22]; overall, the trial had a low risk of bias (File S4).

### Results of individual studies

The impact of self‐financing patient support groups on blood pressure and diabetes parameters is shown in Table 2. Pastakia et al. [21] observed reductions in blood pressure (both systolic and diastolic), with the biggest reduction in systolic blood pressure at 12 months of the intervention (−23 mmHg change from baseline). In Vedanthan et al. [22], a microfinance intervention resulted in a 14.8 mmHg reduction in systolic blood pressure compared with baseline, and a combined group medical visits and microfinance intervention resulted in a 16.4 mmHg reduction at 12 months of the intervention. However, usual care also resulted in an 11.4 mmHg reduction. Compared with usual care, the combined GMV and microfinance intervention resulted in a 3.2 mmHg drop in systolic blood pressure. At 12 months, blood pressure control was achieved in 40.2% of the participants. In Reference [23], the intervention resulted in a 16.9 mmHg in systolic and a 10 mmHg decrease in diastolic blood pressure after 12 months of the intervention in individuals with hypertension at baseline. The intervention also resulted in half of the participants achieving their target blood pressure goals.

**TABLE 2:** Impact of self‐financing patient support groups on blood pressure and diabetes parameters

Study	Diabetes parameters	Blood pressure	Other outcomes
Pastakia et al., 2017 [21]	Baseline HbA1c: 10.8% Random blood sugar: 8.9 mmol/L (160.4 mg/dl) 6 months HbA1c: 10% Random blood sugar: 8.5 mmol/L (153.2 mg/dl)	Baseline Systolic BP: 163 (158, 169) Diastolic BP: 94 (91, 97) 3 months Systolic BP: 157 (152, 162) *p* = 0.04 Change from baseline: = −7 Diastolic BP: 92 (89, 95) *p* = 0.25 Change from baseline: −2 6 months Systolic BP: 152 (147, 157) *p* < 0.01 Change from baseline: −11 Diastolic BP: 89 (87, 91) *p* = 0.04 Change from baseline: −5 9 months Systolic BP: 144 (139, 149) *p* = <0.01 Change from baseline: −19 Diastolic BP: 93 (90, 95) *p* = 0.64 Change from baseline: −1 12 months Systolic BP: 140 (135, 145) *p* < 0.01 Change from baseline: −23 Diastolic BP: 88 (85, 91) *p* < 0.01 Change from baseline: −6	MF activities resulted in total accumulated savings of $6616.85 with dividend interest payments totalling $3120.40
Vedanthan et al., 2021 [22]	Not reported	Baseline systolic BP UC: 155.4 ± 19.9 MF only: 159.0 ± 18.8 GMV only: 156.4 ± 17.5 GMV and MF: 159.1 ± 20.0 Baseline Diastolic BP UC: 91.2 ± 11.9 MF only: 92.3 ± 11.3 GMV only: 92.8 ± 11.1 GMV and MF: 93.8 ± 12.6 Change in systolic BP at 12 months compared with baseline UC: −11.4 (−12.9, −10.0) MF only: −14.8 (−16.4, −13.3) GMV only: −14.7 (16.2, −13.1) GMV and MF: −16.4 (−18.0, −14.7) Change in diastolic BP at 12 months compared with baseline Not reported Change in systolic BP at 12 months compared with usual care MF versus UC: −2.3 (−6.3, 1.7) GMV versus UC: −3.3 (−7.1, 0.5) GMV‐MF versus UC: −3.9 (−7.8, 0.0) Change in diastolic BP at 12 months compared with usual care MF versus UC: −2.4 (−4.1, −0.6) GMV versus UC: −1.6 (−3.4, 0.1) GMV and MF versus UC: −3.2 (−5.1, −1.5) BP control at 12 months achieved in 40.2% of participants	GMV and MF groups had a greater decrease in QRISK3 score (−0.9) compared with UC. Treatment did not affect wealth indices or lipid levels
Taniguchi et al., 2017 [23]	Baseline Fasting blood glucose: 215.9 mg/dl 10% had fasting blood glucose less than 126 mg/dl Mean fasting blood glucose for patients on diabetes medication: 216 mg/dl 6 months Fasting blood glucose: 169.6 mg/dl Mean fasting blood glucose for patients on diabetes medication: 163 mg/dl 12 months Fasting blood glucose: 153.1 mg/dl Mean fasting blood glucose for patients on diabetes medication: 159 mg/dl 18 months Fasting blood glucose: 166.5 mg/dl 24 months Fasting blood glucose: 166.2 mg/dl	Baseline Systolic BP: 130 (118, 146) Diastolic BP: 82 (74, 91) Participants with elevated BP at enrolment: 41.6% (927/2230) 12 months (only for individuals with SBP≥140 and DBP≥90) Change in systolic SBP: −16.9 (−1.2, −22.9) Change in diastolic BP: −10 (−0.7, −12.9) Target BP goal achieved for 51.5% of individuals 18 months Not reported 24 months Desired BP levels (<140/90 mmHg) reached for 65.7% of patients.	

Abbreviations: BP, blood pressure; GMV, group medical visits; HbA1C, glycated haemoglobin; MF, microfinance; QRISK3, cardiovascular risk score; UC, usual care.

Pastakia et al. [21] reported HbA1c and random blood glucose measurements at baseline and at 6 months of the intervention. A reduction was observed for both HbA1c (baseline 10.8%, 6 months 10%) random blood sugar (baseline 8.9 mmol/L, 6 months 8.5 mmol/L) but these changes did not achieve statistical significance. Vedanthan et al. [22] did not report diabetes parameters. In the study of Taniguchi et al. [23] the intervention resulted in a reduction in mean fasting glucose from 216 mg/dl at baseline to 159 mg/dl at 12 months for diabetic patients on medication (*p* < 0.001).

### Results of syntheses

All studies demonstrated an improvement in parameters among the patients at the end of the period of follow‐up. In all studies, the intervention resulted in a reduction in blood pressure. For glycaemic parameters, the reductions (reported by two of the three studies) were more subtle. All studies were of fair quality and the trial had a low risk of bias. The quality of evidence is moderate.

## DISCUSSION

This is the first systematic review, to our knowledge, of the impact of self‐financing patient support groups for diabetes and hypertension on blood pressure and glycaemic parameters. The review has produced two major findings. First, self‐financing patient support groups can improve blood pressure and glycaemic parameters in patients with hypertension and diabetes. Second, evidence on these self‐financing patient groups is scarce as shown by only three articles in the literature.

Our findings are consistent with systematic reviews that have generally (without a focus on self‐financing) looked at the impact of patient support groups on diabetes and hypertension. A systematic review by Werfalli et al. showed that peer and community health worker‐led self‐management support programs improved clinical, behavioural and psychological outcomes in diabetic patients in LMICs [9]. According to a systematic review, group‐directed interventions such as support groups had a significant impact on HbA1c and had more effect than individual directed interventions on blood pressure [24]. A systematic review evaluating the impact of social support on adherence to medications in hypertensive patients showed that peer support may promote better adherence [11].

The strategy of self‐financing groups has been deployed for other health outcomes and this has been through microfinance groups. A systematic review on the impact of microfinance interventions on HIV treatment outcomes showed that they improved treatment adherence, retention in care and viral suppression [25]. In India, women's self‐help groups with a microfinance component resulted in improvements in maternal and newborn health practices [26], child nutrition [27] and mental health [28]. Female sex workers in Tanzania who participated in community (peer) groups with a financial saving component had more consistent condom use with new clients [29]. In addition, a microfinance and support group intervention resulted in low intimate partner violence in the informal settlements of Nairobi Kenya [30]. Integrating microfinance and health interventions such as health micro‐insurance, linkage to health care and access to health products, is beneficial [25, 31, 32, 33].

However, this review revealed the scarcity of evidence on self‐financing patient support groups in the management of hypertension and diabetes. The review identified only three studies in LMICs conducted mainly in rural areas and only one was a randomised controlled trial. Efforts such as the Harambee trial, an ongoing cluster‐randomised trial in Kenya that is evaluating integrated community‐based HIV and non‐communicable disease care incorporated into microfinance groups, are a welcome addition to this field [34]. However, more studies are required to fill this critical gap in the literature. Possible areas to investigate include cost‐effectiveness, more robust evidence on clinical outcomes, the potential to improve linkage to care and whether obligatory payments may increase pressure on individuals and potentially force some patients to drop out of care.

Strengths of our review include the rigorous methods used to develop the review, the addition of grey literature search to the conventional database search, and the inclusion of studies in English and French to minimise language bias. The review had some limitations, which we acknowledge. Owing to the scarcity of evidence, only three studies were included, a retrospective cohort, before‐after study and a randomised controlled trial. Considering that the included studies had different study designs, no meta‐analysis was done. In addition, the review does not disentangle the effectiveness of patient support groups in general from the influence of self‐financing of such groups. This may limit the strength of our conclusions.

In areas with limited health insurance coverage, the self‐financing patient support groups' concept appears to be a promising strategy for managing patients with hypertension and diabetes. However, there are very few studies that have been conducted in these settings and these have not been rigorously evaluated. There is a need to generate more evidence to recognise self‐financing patient support groups as an effective strategy for improving care of patients with diabetes and hypertension in low resource settings.

## FUNDING INFORMATION

This work was supported by the Joint Global Health Scheme with funding from the UK Foreign, Commonwealth and Development Office, the UK Medical Research Council, the UK Department of Health and Social Care through the National Institute of Health Research (NIHR) and Wellcome (Grant no: MR/V004484/1) awarded to Gershim Asiki. The funder was not involved in the design of the review, collection, analysis or interpretation of data, writing of or decision to publish this paper.

## CONFLICT OF INTEREST

The authors declare no conflicts of interest.

## Supporting information


**Data S1:** Supporting Information

## References

[1] World Health Organisation . Hypertension 2021 Available from: https://www.who.int/news-room/fact-sheets/detail/hypertension.

[2] International Diabetes Federation. IDF Diabetes Atlas2019.

[3] Worldwide trends in diabetes since 1980: a pooled analysis of 751 population‐based studies with 4.4 million participants. Lancet. 2016;387(10027):1513–30. 27061677 10.1016/S0140-6736(16)00618-8 PMC5081106

[4] Mills KT , Bundy JD , Kelly TN , Reed JE , Kearney PM , Reynolds K , et al. Global disparities of hypertension prevalence and control: a systematic analysis of population‐based studies from 90 countries. Circulation. 2016;134(6):441–50. 27502908 10.1161/CIRCULATIONAHA.115.018912 PMC4979614

[5] Manne‐Goehler J , Atun R , Stokes A , Goehler A , Houinato D , Houehanou C , et al. Diabetes diagnosis and care in sub‐Saharan Africa: pooled analysis of individual data from 12 countries. Lancet Diabetes Endocrinol. 2016;4(11):903–12. 27727123 10.1016/S2213-8587(16)30181-4

[6] Atun R , Davies JI , Gale EAM , Bärnighausen T , Beran D , Kengne AP , et al. Diabetes in sub‐Saharan Africa: from clinical care to health policy. Lancet Diabetes Endocrinol. 2017;5(8):622–67. 28688818 10.1016/S2213-8587(17)30181-X

[7] de Silva D . Helping people help themselves: a review of the evidence considering whether it is worthwhile to support self‐management. London: The Health Foundation; 2011.

[8] Fisher EB , Boothroyd RI , Elstad EA , Hays L , Henes A , Maslow GR , et al. Peer support of complex health behaviors in prevention and disease management with special reference to diabetes: systematic reviews. Clin Diabetes Endocrinol. 2017;3:4. 28702258 10.1186/s40842-017-0042-3 PMC5471959

[9] Werfalli M , Raubenheimer PJ , Engel M , Musekiwa A , Bobrow K , Peer N , et al. The effectiveness of peer and community health worker‐led self‐management support programs for improving diabetes health‐related outcomes in adults in low‐ and‐middle‐income countries: a systematic review. Syst Rev. 2020;9(1):133. 32505214 10.1186/s13643-020-01377-8 PMC7275531

[10] World Health Organization . Guidance on operations and service delivery. In: Consolidated guidelines on the use of antiretroviral drugs for treating and preventing HIV infection: recommendations for a public health approach. Geneva: World Health Organization; 2013. 24716260

[11] Shahin W , Kennedy GA , Stupans I . The association between social support and medication adherence in patients with hypertension: a systematic review. Pharm Pract (Granada). 2021;19(2):2300. 34221197 10.18549/PharmPract.2021.2.2300 PMC8234709

[12] Mendis S , Fukino K , Cameron A , Laing R , Filipe A Jr , Khatib O , et al. The availability and affordability of selected essential medicines for chronic diseases in six low‐ and middle‐income countries. Bull World Health Organ. 2007;85(4):279–88. 17546309 10.2471/BLT.06.033647 PMC2636320

[13] Cameron A , Ewen M , Ross‐Degnan D , Ball D , Laing R . Medicine prices, availability, and affordability in 36 developing and middle‐income countries: a secondary analysis. Lancet. 2009;373(9659):240–9. 19042012 10.1016/S0140-6736(08)61762-6

[14] Kazungu JS , Barasa EW . Examining levels, distribution and correlates of health insurance coverage in Kenya. Tropical Med Int Health. 2017;22(9):1175–85. 10.1111/tmi.12912 PMC5599961 28627085

[15] Sanya RE , Asiki G , Klipstein‐Grobusch K , Mahone S , Levitt N . Systematic review of self‐financing patient‐led support groups in the management of hypertension and diabetes in low‐ and middle‐income countries. PROSPERO, CRD42021282203. 2021 Available from: https://www.crd.york.ac.uk/prospero/display_record.php?ID=CRD42021282203. 10.1111/tmi.13842 PMC10107175 36518014

[16] Shamseer L , Moher D , Clarke M , Ghersi D , Liberati A , Petticrew M , et al. Preferred reporting items for systematic review and meta‐analysis protocols (PRISMA‐P) 2015: elaboration and explanation. BMJ. 2015;350:g7647. 25555855 10.1136/bmj.g7647

[17] World Bank . World Bank country and lending groups. Washington DC: World Bank Group; 2021. [cited 2021 Aug 18] Available from: https://datahelpdesk.worldbank.org/knowledgebase/articles/906519-world-bank-country-and-lending-groups

[18] OPENGREY.EU – Grey Literature Database. Available from: https://opengrey.eu/.

[19] Ouzzani M , Hammady H , Fedorowicz Z , Elmagarmid A . Rayyan‐a web and mobile app for systematic reviews. Syst Rev. 2016;5(1):210. 27919275 10.1186/s13643-016-0384-4 PMC5139140

[20] National Institutes of Health. National Institutes of Health Quality Assessment tool for observational cohort and cross‐sectional studies 2016 Available from: https://www.nhlbi.nih.gov/health-topics/study-quality-assessment-tools.

[21] Pastakia SD , Manyara SM , Vedanthan R , Kamano JH , Menya D , Andama B , et al. Impact of bridging income generation with group integrated care (BIGPIC) on hypertension and diabetes in rural western Kenya. J Gen Intern Med. 2017;32(5):540–8. 27921256 10.1007/s11606-016-3918-5 PMC5400758

[22] Vedanthan R , Kamano JH , Chrysanthopoulou SA , Mugo R , Andama B , Bloomfield GS , et al. Group medical visit and microfinance intervention for patients with diabetes or hypertension in Kenya. J Am Coll Cardiol. 2021;77(16):2007–18. 33888251 10.1016/j.jacc.2021.03.002 PMC8065205

[23] Taniguchi D , LoGerfo J , van Pelt M , Mielcarek B , Huster K , Haider M , et al. Evaluation of a multi‐faceted diabetes care program including community‐based peer educators in Takeo province, Cambodia, 2007‐2013. PLoS One. 2017;12(9):e0181582. 28945753 10.1371/journal.pone.0181582 PMC5612455

[24] Chen YC , Li IC . Effectiveness of interventions using empowerment concept for patients with chronic disease: a systematic review. JBI Libr Syst Rev. 2009;7(27):1179–233. 27819885 10.11124/01938924-200907270-00001

[25] Nadkarni S , Genberg B , Galárraga O . Microfinance interventions and HIV treatment outcomes: a synthesizing conceptual framework and systematic review. AIDS Behav. 2019;23(9):2238–52. 30805757 10.1007/s10461-019-02443-6 PMC6708758

[26] Hazra A , Atmavilas Y , Hay K , Saggurti N , Verma RK , Ahmad J , et al. Effects of health behaviour change intervention through women's self‐help groups on maternal and newborn health practices and related inequalities in rural India: a quasi‐experimental study. EClinicalMedicine. 2020;18:100198. 31993574 10.1016/j.eclinm.2019.10.011 PMC6978187

[27] Ojha S , Szatkowski L , Sinha R , Yaron G , Fogarty A , Allen SJ , et al. Rojiroti microfinance and child nutrition: a cluster randomised trial. Arch Dis Child. 2020;105(3):229–35. 31601571 10.1136/archdischild-2018-316471 PMC7041497

[28] Mohindra K , Haddad S , Narayana D . Can microcredit help improve the health of poor women? Some findings from a cross‐sectional study in Kerala. Int J Equity Health. 2008;7:2. 18186918 10.1186/1475-9276-7-2 PMC2254417

[29] Mantsios A , Galai N , Mbwambo J , Likindikoki S , Shembilu C , Mwampashi A , et al. Community savings groups, financial security, and HIV risk among female sex workers in Iringa, Tanzania. AIDS Behav. 2018;22(11):3742–50. 29478147 10.1007/s10461-018-2065-x PMC6108953

[30] Sarnquist CC , Ouma L , Lang'at N , Lubanga C , Sinclair J , Baiocchi MT , et al. The effect of combining business training, microfinance, and support group participation on economic status and intimate partner violence in an unplanned settlement of Nairobi, Kenya. J Interpers Violence. 2021;36(7–8):3903–21. 29862883 10.1177/0886260518779067

[31] Leatherman S , Metcalfe M , Geissler K , Dunford C . Integrating microfinance and health strategies: examining the evidence to inform policy and practice. Health Policy Plan. 2012;27(2):85–101. 21343235 10.1093/heapol/czr014

[32] Lorenzetti LMJ , Leatherman S , Flax VL . Evaluating the effect of integrated microfinance and health interventions: an updated review of the evidence. Health Policy Plan. 2017;32(5):732–56. 28453714 10.1093/heapol/czw170

[33] Arrivillaga M , Salcedo JP . A systematic review of microfinance‐based interventions for HIV/AIDS prevention. AIDS Educ Prev. 2014;26(1):13–27. 24450275 10.1521/aeap.2014.26.1.13

[34] Genberg BL , Wachira J , Steingrimsson JA , Pastakia S , Tran DNT , Said JA , et al. Integrated community‐based HIV and non‐communicable disease care within microfinance groups in Kenya: study protocol for the Harambee cluster randomised trial. BMJ Open. 2021;11(5):e042662. 10.1136/bmjopen-2020-042662 PMC8137246 34006540

