# Supplementary material for: Effectiveness of self‐financing patient‐led support groups in the management of hypertension and diabetes in low‐ and middle‐income countries: Systematic review
Source: Trop Med Int Health. 2022 Dec 23;28(2):80–9. doi: 10.1111/tmi.13842 (PMC10107175; doi:10.1111/tmi.13842)
Supplement: Supplementary file 1 — Data S1: Supporting Information [file TMI-28-80-s001.zip › 5. Online supplemental file 2_Search strategy.pdf]

Self-financing patient-led support groups in the management of hypertension and diabetes in low- and middle-income countries: systematic review search strategy

MEDLINE (PubMed)						
Diabetes OR hypertension		Patient support groups		Financial component		Low and Middle Income Countries (LMICs)
("hypertension"[MeSH] OR hypertension[tiab] OR hypertensive[tiab] OR “high blood pressure”[tiab] OR “blood pressure” OR diabetes[tiab] OR diabet*[tiab] OR dm2[tiab] OR niddm[tiab] OR dm 2[tiab] OR t2d[tiab] OR “dm type 2”[tiab] OR “dm type II”[tiab] OR dm1[tiab] OR iddm[tiab] OR dm 1[tiab] OR t1d[tiab] OR “dm type 1”[tiab] OR “dm type I”[tiab] OR "Diabetes Mellitus"[Mesh])	AND	(“support group*”[tiab] OR buddy[tiab] OR “self-help group*” [tiab] OR “peer group*” [tiab] OR “informal group*”[tiab] OR “social group”[tiab] OR “Volunteers"[Mesh] OR “Self-help groups"[Mesh] OR “Peer group"[Mesh] OR "peer/microfinance"[tiab] OR "group medical visits"[tiab])	AND	(financ*[tiab] OR cash[tiab] OR fund[tiab] OR contribution[tiab] OR support[tiab] OR backing[tiab] OR aid[tiab] OR microfinance[tiab])	AND	(“emerging country”[all fields] OR “emerging countries”[all fields] OR “emerging nation”[all fields] OR “emerging nations”[all fields] OR “emerging population”[all fields] OR “emerging populations”[all fields] OR "developing country"[tiab] OR "developing countries"[tiab] OR "developing nation"[tiab] OR "developing nations"[tiab] OR "developing population"[tiab] OR "developing populations"[tiab] OR "developing world"[tiab] OR "less developed country"[tiab] OR "less developed countries"[tiab] OR "less developed nation"[tiab] OR "less developed nations"[tiab] OR "less developed world"[tiab] OR "lesser developed countries"[tiab] OR "lesser developed nations"[tiab] OR "under developed country"[tiab] OR "under developed countries"[tiab] OR "under developed nations"[tiab] OR "under developed world"[tiab] OR "underdeveloped country"[tiab] OR "underdeveloped countries"[tiab] OR "underdeveloped nation"[tiab] OR "underdeveloped nations"[tiab] OR "underdeveloped population"[tiab] OR "underdeveloped populations"[tiab] OR "underdeveloped world"[tiab] OR "middle income country"[tiab] OR "middle income countries"[tiab] OR "middle income nation"[tiab] OR "middle income nations"[tiab] OR "middle income population"[tiab] OR "middle income populations"[tiab] OR "low income country"[tiab] OR "low income countries"[tiab] OR "low income nation"[tiab] OR "low income nations"[tiab] OR "low income population"[tiab] OR "low income populations"[tiab] OR "lower income country"[tiab] OR "lower income countries"[tiab] OR "lower income nations"[tiab] OR "lower income population"[tiab] OR "lower income populations"[tiab] OR "underserved countries"[tiab] OR "underserved nations"[tiab] OR "underserved population"[tiab] OR "underserved populations"[tiab] OR "under served population"[tiab] OR "under served populations"[tiab] OR "deprived countries"[tiab] OR "deprived population"[tiab] OR "deprived populations"[tiab] OR "poor country"[tiab] OR "poor countries"[tiab] OR "poor nation"[tiab] OR "poor nations"[tiab] OR "poor population"[tiab] OR "poor populations"[tiab] OR "poor world"[tiab] OR "poorer countries"[tiab] OR "poorer nations"[tiab] OR "poorer population"[tiab] OR "poorer populations"[tiab] OR "developing economy"[tiab] OR "developing economies"[tiab] OR "less developed economy"[tiab] OR "less developed economies"[tiab] OR "underdeveloped economies"[tiab] OR "middle income economy"[tiab] OR "middle income economies"[tiab] OR "low income economy"[tiab] OR "low income economies"[tiab] OR "lower income economies"[tiab] OR "low gdp"[tiab] OR "low gnp"[tiab] OR "low gross domestic"[tiab] OR "low gross national"[tiab] OR "lower gdp"[tiab] OR "lower gross domestic"[tiab] OR lmic[tiab] OR lmics[tiab] OR "third world"[tiab] OR "lami country"[tiab] OR "lami countries"[tiab] OR "transitional country"[tiab] OR "transitional countries"[tiab] OR Africa[tiab] OR Asia[tiab] OR Caribbean[tiab] OR West Indies[tiab] OR South America[tiab] OR Latin America[tiab] OR Central America[tiab] OR "Atlantic Islands"[tiab] OR "Pacific Islands"[tiab] OR "Indian Ocean Islands"[tiab] OR Afghanistan[tiab] OR Albania[tiab] OR Algeria[tiab] OR Angola[tiab] OR Argentina[tiab] OR Armenia[tiab] OR Azerbaijan[tiab] OR Bangladesh[tiab] OR Barbados[tiab] OR Benin[tiab] OR Byelarus[tiab] OR Byelorussian[tiab] OR Belarus[tiab] OR Belorussian[tiab] OR Belorussia[tiab] OR Belize[tiab] OR Bhutan[tiab] OR Bolivia[tiab] OR Bosnia[tiab] OR Herzegovina[tiab] OR Hercegovina[tiab] OR Botswana[tiab] OR Brasil[tiab] OR Brazil[tiab] OR Bulgaria[tiab] OR Burkina Faso[tiab] OR Burkina Fasso[tiab] OR Upper Volta[tiab] OR Burundi[tiab] OR Urundi[tiab] OR Cambodia[tiab] OR Khmer Republic[tiab] OR Kampuchea[tiab] OR Cameroon[tiab] OR Cameroons[tiab] OR Cameron[tiab] OR Cape Verde[tiab] OR Central African Republic[tiab] OR Chad[tiab] OR China[tiab] OR Colombia[tiab] OR Comoros[tiab] OR Comoro Islands[tiab] OR Comores[tiab] OR Mayotte[tiab] OR Congo[tiab] OR Zaire[tiab]

						OR Costa Rica[tiab] OR Cote d'Ivoire[tiab] OR Ivory Coast[tiab] OR Cuba[tiab] OR Djibouti[tiab] OR French Somaliland[tiab] OR Dominica[tiab] OR Dominican Republic[tiab] OR East Timor[tiab] OR East Timur[tiab] OR Timor Leste[tiab] OR Ecuador[tiab] OR Egypt[tiab] OR El Salvador[tiab] OR Eritrea[tiab] OR Ethiopia[tiab] OR Fiji[tiab] OR Gabon[tiab] OR Gabonese Republic[tiab] OR Gambia[tiab] OR Gaza[tiab] OR Georgia[tiab] OR Ghana[tiab] OR Gold Coast[tiab] OR Grenada[tiab] OR Guatemala[tiab] OR Guinea[tiab] OR Guam[tiab] OR Guiana[tiab] OR Guyana[tiab] OR Haiti[tiab] OR Honduras[tiab] OR India[tiab] OR Indonesia[tiab] OR Iran[tiab] OR Iraq[tiab] OR Jamaica[tiab] OR Jordan[tiab] OR Kazakhstan[tiab] OR Kenya[tiab] OR Kiribati[tiab] OR Kosovo[tiab] OR Kyrgyzstan[tiab] OR Kirghizia[tiab] OR Kyrgyz Republic[tiab] OR Kirghiz[tiab] OR Kirgizstan[tiab] OR "Lao PDR"[tiab] OR Laos[tiab] OR Latvia[tiab] OR Lebanon[tiab] OR Lesotho[tiab] OR Liberia[tiab] OR Libya[tiab] OR Macedonia[tiab] OR Madagascar[tiab] OR Malaysia[tiab] OR Malaya[tiab] OR Malay[tiab] OR Maldives[tiab] OR Malawi[tiab] OR Mali[tiab] OR Marshall Islands[tiab] OR Mauritania[tiab] OR Mauritius[tiab] OR Mexico[tiab] OR Micronesia[tiab] OR Moldova[tiab] OR Moldovia[tiab] OR Mongolia[tiab] OR Montenegro[tiab] OR Morocco[tiab] OR Mozambique[tiab] OR Myanmar[tiab] OR Myanma[tiab] OR Burma[tiab] OR Namibia[tiab] OR Nepal[tiab] OR Nicaragua[tiab] OR Niger[tiab] OR Nigeria[tiab] OR Pakistan[tiab] OR Panama[tiab] OR Paraguay[tiab] OR Peru[tiab] OR Philippines[tiab] OR Philipines[tiab] OR Phillipines[tiab] OR Romania[tiab] OR Rumania[tiab] OR Roumania[tiab] OR Russia[tiab] OR Russian[tiab] OR Rwanda[tiab] OR Ruanda[tiab] OR Saint Lucia[tiab] OR St Lucia[tiab] OR Saint Vincent[tiab] OR St Vincent[tiab] OR Grenadines[tiab] OR Samoa[tiab] OR Sao Tome[tiab] OR Senegal[tiab] OR Serbia[tiab] OR Montenegro[tiab] OR Sierra Leone[tiab] OR Sri Lanka[tiab] OR Ceylon[tiab] OR Solomon Islands[tiab] OR Somalia[tiab] OR Sudan[tiab] OR Suriname[tiab] OR Surinam[tiab] OR Swaziland[tiab] OR Eswatini[tiab] OR South Africa OR Syria[tiab] OR Tajikistan[tiab] OR Tadzhikistan[tiab] OR Tadjikistan[tiab] OR Tanzania[tiab] OR Thailand[tiab] OR Togo[tiab] OR Tonga[tiab] OR Tunisia[tiab] OR Turkey[tiab] OR Turkmenistan[tiab] OR Tuvalu[tiab] OR Uganda[tiab] OR Ukraine[tiab] OR Russia[tiab] OR USSR[tiab] OR Soviet Union[tiab] OR Uzbekistan[tiab] OR Uzbek OR Vanuatu[tiab] OR Venezuela[tiab] OR Vietnam[tiab] OR Viet Nam[tiab] OR West Bank[tiab] OR Yemen[tiab] OR Zambia[tiab] OR Zimbabwe[tiab] OR Developing Countries[Mesh] OR Africa[Mesh:NoExp] OR Africa, Northern[Mesh:NoExp] OR Africa South of the Sahara[Mesh:NoExp] OR Africa, Central[Mesh:NoExp] OR Africa, Eastern[Mesh:NoExp] OR Africa, Southern[Mesh:NoExp] OR Africa, Western[Mesh:NoExp] OR Asia[Mesh:NoExp] OR Asia, Central[Mesh:NoExp] OR Asia, Southeastern[Mesh:NoExp] OR Asia, Western[Mesh:NoExp] OR Caribbean Region[Mesh:NoExp] OR West Indies[Mesh:NoExp] OR South America[Mesh:NoExp] OR Latin America[Mesh:NoExp] OR Central America[Mesh:NoExp] OR "Atlantic Islands"[Mesh:NoExp] OR "Pacific Islands"[Mesh:NoExp] OR "Indian Ocean Islands"[Mesh:NoExp] OR Afghanistan[Mesh] OR Albania[Mesh] OR Algeria[Mesh] OR American Samoa[Mesh] OR Angola[Mesh] OR Argentina[Mesh] OR Armenia[Mesh] OR Azerbaijan[Mesh] OR Bangladesh[Mesh] OR Barbados[Mesh] OR Benin[Mesh] OR "Republic of Belarus"[Mesh] OR Belize[Mesh] OR Bhutan[Mesh] OR Bolivia[Mesh] OR Bosnia-Herzegovina[Mesh] OR Botswana[Mesh] OR Brazil[Mesh] OR Bulgaria[Mesh] OR Burkina Faso[Mesh] OR Burundi[Mesh] OR Cambodia[Mesh] OR Cameroon[Mesh] OR Cape Verde[Mesh] OR Central African Republic[Mesh] OR Chad[Mesh] OR China[Mesh] OR Colombia[Mesh] OR Comoros[Mesh] OR Congo[Mesh] OR Costa Rica[Mesh] OR Cote d'Ivoire[Mesh] OR Croatia[Mesh] OR Cuba[Mesh] OR Slovakia[Mesh] OR Djibouti[Mesh] OR "Democratic Republic of the Congo"[Mesh] OR Dominica[Mesh] OR Dominican Republic[Mesh] OR East Timor[Mesh] OR Ecuador[Mesh] OR Egypt[Mesh] OR El Salvador[Mesh] OR Eritrea[Mesh] OR Ethiopia[Mesh] OR "Equatorial Guinea"[Mesh] OR Fiji[Mesh] OR "French Guiana"[Mesh] OR Gabon[Mesh] OR Gambia[Mesh] OR Ghana[Mesh] OR Greece[Mesh] OR Grenada[Mesh] OR Guatemala[Mesh] OR Guinea[Mesh] OR Guinea-Bissau[Mesh] OR Guam[Mesh] OR Guyana[Mesh] OR Haiti[Mesh] OR Honduras[Mesh] OR "Independent State of Samoa"[Mesh] OR India[Mesh] OR Indonesia[Mesh] OR Iran[Mesh] OR Iraq[Mesh] OR Jamaica[Mesh] OR Jordan[Mesh] OR Kazakhstan[Mesh] OR Kenya[Mesh] OR Korea[Mesh] OR

						Kyrgyzstan[Mesh] OR Laos[Mesh] OR Latvia[Mesh] OR Lebanon[Mesh] OR Lesotho[Mesh] OR Liberia[Mesh] OR Libya[Mesh] OR "Macedonia"[Mesh] OR Madagascar[Mesh] OR Malawi[Mesh] OR Malaysia[Mesh] OR Mali[Mesh] OR Malta[Mesh] OR Mauritania[Mesh] OR Mauritius[Mesh] OR "Melanesia"[Mesh] OR Mexico[Mesh] OR Micronesia[Mesh] OR Moldova[Mesh] OR Mongolia[Mesh] OR Montenegro[Mesh] OR Morocco[Mesh] OR Mozambique[Mesh] OR Myanmar[Mesh] OR Namibia[Mesh] OR Nepal[Mesh] OR Nicaragua[Mesh] OR Niger[Mesh] OR Nigeria[Mesh] OR Pakistan[Mesh] OR Panama[Mesh] OR Papua New Guinea[Mesh] OR Paraguay[Mesh] OR Peru[Mesh] OR Philippines[Mesh] OR "Republic of Korea"[Mesh] OR Romania[Mesh] OR Russia[Mesh] OR "Russia (Pre-1917)"[Mesh] OR Rwanda[Mesh] OR Saint Lucia[Mesh] OR "Saint Vincent and the Grenadines"[Mesh] OR Samoa[Mesh] OR Senegal[Mesh] OR Serbia[Mesh] OR Montenegro[Mesh] OR Sierra Leone[Mesh] OR Slovenia[Mesh] OR Sri Lanka[Mesh] OR Somalia[Mesh] OR South Africa[Mesh] OR Sudan[Mesh] OR Suriname[Mesh] OR Swaziland[Mesh] OR Syria[Mesh] OR Tajikistan[Mesh] OR Tanzania[Mesh] OR Thailand[Mesh] OR Togo[Mesh] OR Tonga[Mesh] OR Tunisia[Mesh] OR Turkey[Mesh] OR Turkmenistan[Mesh] OR Uganda[Mesh] OR Ukraine[Mesh] OR Uruguay[Mesh] OR USSR[Mesh] OR Uzbekistan[Mesh] OR Vanuatu[Mesh] OR Venezuela[Mesh] OR Vietnam[Mesh] OR Yemen[Mesh] OR Yugoslavia[Mesh] OR Zambia[Mesh] OR Zimbabwe[Mesh] OR “Sub Saharan Africa "[all fields] OR “SubSaharan Africa "[all fields])
**Combined search syntax**						
("hypertension"[MeSH] OR hypertension[tiab] OR hypertensive[tiab] OR “high blood pressure”[tiab] OR “blood pressure” OR diabetes[tiab] OR diabet*[tiab] OR dm2[tiab] OR niddm[tiab] OR dm 2[tiab] OR t2d[tiab] OR “dm type 2”[tiab] OR “dm type II”[tiab] OR dm1[tiab] OR iddm[tiab] OR dm 1[tiab] OR t1d[tiab] OR “dm type 1”[tiab] OR “dm type I”[tiab] OR "Diabetes Mellitus"[Mesh]) AND (“support group*”[tiab] OR buddy[tiab] OR “self-help group*” [tiab] OR “peer group*” [tiab] OR “informal group*”[tiab] OR “social group”[tiab] OR “Volunteers"[Mesh] OR “Self-help groups"[Mesh] OR “Peer group"[Mesh] OR "peer microfinance"[tiab] OR "group medical visits"[tiab]) AND (financ*[tiab] OR cash[tiab] OR fund[tiab] OR contribution[tiab] OR support[tiab] OR backing[tiab] OR aid[tiab] OR microfinance[tiab]) AND (“emerging country”[all fields] OR “emerging countries”[all fields] OR “emerging nation”[all fields] OR “emerging nations”[all fields] OR “emerging population”[all fields] OR “emerging populations”[all fields] OR "developing country"[tiab] OR "developing countries"[tiab] OR "developing nation"[tiab] OR "developing nations"[tiab] OR "developing population"[tiab] OR "developing populations"[tiab] OR "developing world"[tiab] OR "less developed country"[tiab] OR "less developed countries"[tiab] OR "less developed nation"[tiab] OR "less developed nations"[tiab] OR "less developed world"[tiab] OR "lesser developed countries"[tiab] OR "lesser developed nations"[tiab] OR "under developed country"[tiab] OR "under developed countries"[tiab] OR "under developed nations"[tiab] OR "under developed world"[tiab] OR "underdeveloped country"[tiab] OR "underdeveloped countries"[tiab] OR "underdeveloped nation"[tiab] OR "underdeveloped nations"[tiab] OR "underdeveloped population"[tiab] OR "underdeveloped populations"[tiab] OR "underdeveloped world"[tiab] OR "middle income country"[tiab] OR "middle income countries"[tiab] OR "middle income nation"[tiab] OR "middle income nations"[tiab] OR "middle income population"[tiab] OR "middle income populations"[tiab] OR "low income country"[tiab] OR "low income countries"[tiab] OR "low income nation"[tiab] OR "low income nations"[tiab] OR "low income population"[tiab] OR "low income populations"[tiab] OR "lower income country"[tiab] OR "lower income countries"[tiab] OR "lower income nations"[tiab] OR "lower income population"[tiab] OR "lower income populations"[tiab] OR "underserved countries"[tiab] OR "underserved nations"[tiab] OR "underserved population"[tiab] OR "underserved populations"[tiab] OR "under served population"[tiab] OR "under served populations"[tiab] OR "deprived countries"[tiab] OR "deprived population"[tiab] OR "deprived populations"[tiab] OR "poor country"[tiab] OR "poor countries"[tiab] OR "poor nation"[tiab] OR "poor nations"[tiab] OR "poor population"[tiab] OR "poor populations"[tiab] OR "poor world"[tiab] OR "poorer countries"[tiab] OR "poorer nations"[tiab] OR "poorer population"[tiab] OR "poorer populations"[tiab] OR "developing economy"[tiab] OR "developing economies"[tiab] OR "less developed economy"[tiab] OR "less developed economies"[tiab] OR "underdeveloped economies"[tiab] OR "middle income economy"[tiab] OR "middle income economies"[tiab] OR "low income economy"[tiab] OR "low income economies"[tiab] OR "lower income economies"[tiab] OR "low gdp"[tiab] OR "low gnp"[tiab] OR "low gross domestic"[tiab] OR "low gross national"[tiab] OR "lower gdp"[tiab] OR "lower gross domestic"[tiab] OR lmic[tiab] OR lmics[tiab] OR "third world"[tiab] OR "lami country"[tiab] OR "lami countries"[tiab] OR "transitional country"[tiab] OR "transitional countries"[tiab] OR Africa[tiab] OR Asia[tiab] OR Caribbean[tiab] OR West Indies[tiab] OR South America[tiab] OR Latin America[tiab] OR Central America[tiab] OR "Atlantic Islands"[tiab] OR "Pacific Islands"[tiab] OR "Indian Ocean Islands"[tiab] OR Afghanistan[tiab] OR Albania[tiab] OR Algeria[tiab] OR Angola[tiab] OR Argentina[tiab] OR Armenia[tiab] OR Azerbaijan[tiab] OR Bangladesh[tiab] OR Barbados[tiab] OR Benin[tiab] OR Byelarus[tiab] OR Byelorussian[tiab] OR Belarus[tiab] OR Belorussian[tiab] OR Belorussia[tiab] OR Belize[tiab] OR Bhutan[tiab] OR Bolivia[tiab] OR Bosnia[tiab] OR Herzegovina[tiab] OR Hercegovina[tiab] OR Botswana[tiab] OR Brasil[tiab] OR Brazil[tiab] OR Bulgaria[tiab] OR Burkina Faso[tiab] OR Burkina Fasso[tiab] OR Upper Volta[tiab] OR Burundi[tiab] OR Urundi[tiab] OR Cambodia[tiab] OR Khmer Republic[tiab] OR Kampuchea[tiab] OR Cameroon[tiab] OR Cameroons[tiab] OR Cameron[tiab] OR Cape Verde[tiab] OR Central African Republic[tiab] OR Chad[tiab] OR China[tiab] OR Colombia[tiab] OR Comoros[tiab] OR Comoro Islands[tiab] OR Comores[tiab] OR Mayotte[tiab] OR Congo[tiab] OR Zaire[tiab] OR Costa Rica[tiab] OR Cote d'Ivoire[tiab] OR Ivory Coast[tiab] OR Cuba[tiab] OR Djibouti[tiab] OR French Somaliland[tiab] OR Dominica[tiab] OR Dominican Republic[tiab] OR East Timor[tiab] OR East Timur[tiab] OR Timor Leste[tiab] OR Ecuador[tiab] OR Egypt[tiab] OR El Salvador[tiab] OR Eritrea[tiab] OR Ethiopia[tiab] OR Fiji[tiab] OR Gabon[tiab] OR Gabonese Republic[tiab] OR Gambia[tiab] OR Gaza[tiab] OR Georgia[tiab] OR Ghana[tiab] OR Gold Coast[tiab] OR Grenada[tiab] OR Guatemala[tiab] OR Guinea[tiab] OR Guam[tiab] OR Guiana[tiab] OR Guyana[tiab] OR Haiti[tiab] OR Honduras[tiab] OR India[tiab] OR Indonesia[tiab] OR Iran[tiab] OR Iraq[tiab] OR Jamaica[tiab] OR Jordan[tiab] OR Kazakhstan[tiab] OR Kenya[tiab] OR Kiribati[tiab] OR Kosovo[tiab] OR Kyrgyzstan[tiab] OR Kirghizia[tiab] OR Kyrgyz Republic[tiab] OR Kirghiz[tiab] OR Kirgizstan[tiab] OR "Lao PDR"[tiab] OR Laos[tiab] OR						

Latvia[tiab] OR Lebanon[tiab] OR Lesotho[tiab] OR Liberia[tiab] OR Libya[tiab] OR Macedonia[tiab] OR Madagascar[tiab] OR Malaysia[tiab] OR Malaya[tiab] OR Malay[tiab] OR Maldives[tiab] OR Malawi[tiab] OR Mali[tiab] OR Marshall Islands[tiab] OR Mauritania[tiab] OR Mauritius[tiab] OR Mexico[tiab] OR Micronesia[tiab] OR Moldova[tiab] OR Moldovia[tiab] OR Mongolia[tiab] OR Montenegro[tiab] OR Morocco[tiab] OR Mozambique[tiab] OR Myanmar[tiab] OR Myanma[tiab] OR Burma[tiab] OR Namibia[tiab] OR Nepal[tiab] OR Nicaragua[tiab] OR Niger[tiab] OR Nigeria[tiab] OR Pakistan[tiab] OR Panama[tiab] OR Paraguay[tiab] OR Peru[tiab] OR Philippines[tiab] OR Philipines[tiab] OR Phillipines[tiab] OR Romania[tiab] OR Rumania[tiab] OR Roumania[tiab] OR Russia[tiab] OR Russian[tiab] OR Rwanda[tiab] OR Ruanda[tiab] OR Saint Lucia[tiab] OR St Lucia[tiab] OR Saint Vincent[tiab] OR St Vincent[tiab] OR Grenadines[tiab] OR Samoa[tiab] OR Sao Tome[tiab] OR Senegal[tiab] OR Serbia[tiab] OR Montenegro[tiab] OR Sierra Leone[tiab] OR Sri Lanka[tiab] OR Ceylon[tiab] OR Solomon Islands[tiab] OR Somalia[tiab] OR Sudan[tiab] OR Suriname[tiab] OR Surinam[tiab] OR Swaziland[tiab] OR Eswatini[tiab] OR South Africa OR Syria[tiab] OR Tajikistan[tiab] OR Tadzhikistan[tiab] OR Tadjikistan[tiab] OR Tanzania[tiab] OR Thailand[tiab] OR Togo[tiab] OR Tonga[tiab] OR Tunisia[tiab] OR Turkey[tiab] OR Turkmenistan[tiab] OR Tuvalu[tiab] OR Uganda[tiab] OR Ukraine[tiab] OR Russia[tiab] OR USSR[tiab] OR Soviet Union[tiab] OR Uzbekistan[tiab] OR Uzbek OR Vanuatu[tiab] OR Venezuela[tiab] OR Vietnam[tiab] OR Viet Nam[tiab] OR West Bank[tiab] OR Yemen[tiab] OR Zambia[tiab] OR Zimbabwe[tiab] OR Developing Countries[Mesh] OR Africa[Mesh:NoExp] OR Africa, Northern[Mesh:NoExp] OR Africa South of the Sahara[Mesh:NoExp] OR Africa, Central[Mesh:NoExp] OR Africa, Eastern[Mesh:NoExp] OR Africa, Southern[Mesh:NoExp] OR Africa, Western[Mesh:NoExp] OR Asia[Mesh:NoExp] OR Asia, Central[Mesh:NoExp] OR Asia, Southeastern[Mesh:NoExp] OR Asia, Western[Mesh:NoExp] OR Caribbean Region[Mesh:NoExp] OR West Indies[Mesh:NoExp] OR South America[Mesh:NoExp] OR Latin America[Mesh:NoExp] OR Central America[Mesh:NoExp] OR "Atlantic Islands"[Mesh:NoExp] OR "Pacific Islands"[Mesh:NoExp] OR "Indian Ocean Islands"[Mesh:NoExp] OR Afghanistan[Mesh] OR Albania[Mesh] OR Algeria[Mesh] OR American Samoa[Mesh] OR Angola[Mesh] OR Argentina[Mesh] OR Armenia[Mesh] OR Azerbaijan[Mesh] OR Bangladesh[Mesh] OR Barbados[Mesh] OR Benin[Mesh] OR "Republic of Belarus"[Mesh] OR Belize[Mesh] OR Bhutan[Mesh] OR Bolivia[Mesh] OR Bosnia-Herzegovina[Mesh] OR Botswana[Mesh] OR Brazil[Mesh] OR Bulgaria[Mesh] OR Burkina Faso[Mesh] OR Burundi[Mesh] OR Cambodia[Mesh] OR Cameroon[Mesh] OR Cape Verde[Mesh] OR Central African Republic[Mesh] OR Chad[Mesh] OR China[Mesh] OR Colombia[Mesh] OR Comoros[Mesh] OR Congo[Mesh] OR Costa Rica[Mesh] OR Cote d'Ivoire[Mesh] OR Croatia[Mesh] OR Cuba[Mesh] OR Slovakia[Mesh] OR Djibouti[Mesh] OR "Democratic Republic of the Congo"[Mesh] OR Dominica[Mesh] OR Dominican Republic[Mesh] OR East Timor[Mesh] OR Ecuador[Mesh] OR Egypt[Mesh] OR El Salvador[Mesh] OR Eritrea[Mesh] OR Ethiopia[Mesh] OR "Equatorial Guinea"[Mesh] OR Fiji[Mesh] OR "French Guiana"[Mesh] OR Gabon[Mesh] OR Gambia[Mesh] OR Ghana[Mesh] OR Greece[Mesh] OR Grenada[Mesh] OR Guatemala[Mesh] OR Guinea[Mesh] OR Guinea-Bissau[Mesh] OR Guam[Mesh] OR Guyana[Mesh] OR Haiti[Mesh] OR Honduras[Mesh] OR "Independent State of Samoa"[Mesh] OR India[Mesh] OR Indonesia[Mesh] OR Iran[Mesh] OR Iraq[Mesh] OR Jamaica[Mesh] OR Jordan[Mesh] OR Kazakhstan[Mesh] OR Kenya[Mesh] OR Korea[Mesh] OR Kyrgyzstan[Mesh] OR Laos[Mesh] OR Latvia[Mesh] OR Lebanon[Mesh] OR Lesotho[Mesh] OR Liberia[Mesh] OR Libya[Mesh] OR "Macedonia"[Mesh] OR Madagascar[Mesh] OR Malawi[Mesh] OR Malaysia[Mesh] OR Mali[Mesh] OR Malta[Mesh] OR Mauritania[Mesh] OR Mauritius[Mesh] OR "Melanesia"[Mesh] OR Mexico[Mesh] OR Micronesia[Mesh] OR Moldova[Mesh] OR Mongolia[Mesh] OR Montenegro[Mesh] OR Morocco[Mesh] OR Mozambique[Mesh] OR Myanmar[Mesh] OR Namibia[Mesh] OR Nepal[Mesh] OR Nicaragua[Mesh] OR Niger[Mesh] OR Nigeria[Mesh] OR Pakistan[Mesh] OR Panama[Mesh] OR Papua New Guinea[Mesh] OR Paraguay[Mesh] OR Peru[Mesh] OR Philippines[Mesh] OR "Republic of Korea"[Mesh] OR Romania[Mesh] OR Russia[Mesh] OR "Russia (Pre-1917)"[Mesh] OR Rwanda[Mesh] OR Saint Lucia[Mesh] OR "Saint Vincent and the Grenadines"[Mesh] OR Samoa[Mesh] OR Senegal[Mesh] OR Serbia[Mesh] OR Montenegro[Mesh] OR Sierra Leone[Mesh] OR Slovenia[Mesh] OR Sri Lanka[Mesh] OR Somalia[Mesh] OR South Africa[Mesh] OR Sudan[Mesh] OR Suriname[Mesh] OR Swaziland[Mesh] OR Syria[Mesh] OR Tajikistan[Mesh] OR Tanzania[Mesh] OR Thailand[Mesh] OR Togo[Mesh] OR Tonga[Mesh] OR Tunisia[Mesh] OR Turkey[Mesh] OR Turkmenistan[Mesh] OR Uganda[Mesh] OR Ukraine[Mesh] OR Uruguay[Mesh] OR USSR[Mesh] OR Uzbekistan[Mesh] OR Vanuatu[Mesh] OR Venezuela[Mesh] OR Vietnam[Mesh] OR Yemen[Mesh] OR Yugoslavia[Mesh] OR Zambia[Mesh] OR Zimbabwe[Mesh] OR “Sub Saharan Africa "[all fields] OR “SubSaharan Africa "[all fields])						
**Embase**						
Diabetes OR hypertension		Patient support groups		Financial component		Low and Middle Income Countries (LMICs)
(‘hypertension’/de OR hypertension:ti,ab OR hypertensive:ti,ab OR ‘high blood pressure’:ti,ab OR ‘blood pressure’ OR diabetes:ti,ab OR diabet*:ti,ab OR dm2:ti,ab OR niddm:ti,ab OR dm 2:ti,ab OR t2d:ti,ab OR ‘dm type 2’:ti,ab OR ‘dm type II’:ti,ab OR dm1:ti,ab OR iddm:ti,ab OR dm	AND	(‘support group*’:ti,ab OR buddy:ti,ab OR ‘self-help group*’:ti,ab OR ‘peer group*’:ti,ab OR ‘informal group*’:ti,ab OR ‘social group’:ti,ab OR ‘Volunteers’/de OR ‘Self-help groups’/de OR ‘Peer group’/de OR 'peer/microfinance':tiab OR 'group medical visits':ti,ab)	AND	(financ*:ti,ab OR cash:ti,ab OR fund:ti,ab OR contribution:ti,ab OR support:ti,ab OR backing:ti,ab OR aid:ti,ab OR microfinance:ti,ab)	AND	('emerging country' OR 'emerging countries' OR 'emerging nation' OR 'emerging nations' OR 'emerging population' OR 'emerging populations' OR 'developing country':ti,ab OR 'developing countries':ti,ab OR 'developing nation':ti,ab OR 'developing nations':ti,ab OR 'developing population':ti,ab OR 'developing populations':ti,ab OR 'developing world':ti,ab OR 'less developed country':ti,ab OR 'less developed countries':ti,ab OR 'less developed nation':ti,ab OR 'less developed nations':ti,ab OR 'less developed world':ti,ab OR 'lesser developed countries':ti,ab OR 'lesser developed nations':ti,ab OR 'under developed country':ti,ab OR 'under developed countries':ti,ab OR 'under developed nations':ti,ab OR 'under developed world':ti,ab OR 'underdeveloped country':ti,ab OR 'underdeveloped countries':ti,ab OR 'underdeveloped nation':ti,ab OR 'underdeveloped nations':ti,ab OR 'underdeveloped population':ti,ab OR 'underdeveloped populations':ti,ab OR 'underdeveloped world':ti,ab OR 'middle income country':ti,ab OR 'middle income countries':ti,ab OR 'middle income nation':ti,ab OR 'middle income nations':ti,ab OR 'middle income population':ti,ab OR 'middle income populations':ti,ab OR 'low income country':ti,ab OR 'low income countries':ti,ab OR 'low income nation':ti,ab OR 'low income nations':ti,ab OR 'low income population':ti,ab OR 'low income populations':ti,ab OR 'lower income country':ti,ab OR 'lower income countries':ti,ab OR 'lower income nations':ti,ab OR 'lower income population':ti,ab OR 'lower income populations':ti,ab OR

1:ti,ab OR t1d:ti,ab OR ‘dm type 1’:ti,ab OR ‘dm type I’:ti,ab OR ‘Diabetes Mellitus’/de)						'underserved countries':ti,ab OR 'underserved nations':ti,ab OR 'underserved population':ti,ab OR 'underserved populations':ti,ab OR 'under served population':ti,ab OR 'under served populations':ti,ab OR 'deprived countries':ti,ab OR 'deprived population':ti,ab OR 'deprived populations':ti,ab OR 'poor country':ti,ab OR 'poor countries':ti,ab OR 'poor nation':ti,ab OR 'poor nations':ti,ab OR 'poor population':ti,ab OR 'poor populations':ti,ab OR 'poor world':ti,ab OR 'poorer countries':ti,ab OR 'poorer nations':ti,ab OR 'poorer population':ti,ab OR 'poorer populations':ti,ab OR 'developing economy':ti,ab OR 'developing economies':ti,ab OR 'less developed economy':ti,ab OR 'less developed economies':ti,ab OR 'underdeveloped economies':ti,ab OR 'middle income economy':ti,ab OR 'middle income economies':ti,ab OR 'low income economy':ti,ab OR 'low income economies':ti,ab OR 'lower income economies':ti,ab OR 'low gdp':ti,ab OR 'low gnp':ti,ab OR 'low gross domestic':ti,ab OR 'low gross national':ti,ab OR 'lower gdp':ti,ab OR 'lower gross domestic':ti,ab OR lmic:ti,ab OR lmics:ti,ab OR 'third world':ti,ab OR 'lami country':ti,ab OR 'lami countries':ti,ab OR 'transitional country':ti,ab OR 'transitional countries':ti,ab OR africa:ti,ab OR asia:ti,ab OR caribbean:ti,ab OR 'west indies':ti,ab OR 'south america':ti,ab OR 'latin america':ti,ab OR 'central america':ti,ab OR 'atlantic islands':ti,ab OR 'pacific islands':ti,ab OR 'indian ocean islands':ti,ab OR afghanistan:ti,ab OR albania:ti,ab OR algeria:ti,ab OR angola:ti,ab OR argentina:ti,ab OR armenia:ti,ab OR azerbaijan:ti,ab OR bangladesh:ti,ab OR barbados:ti,ab OR benin:ti,ab OR byelarus:ti,ab OR byelorussian:ti,ab OR belarus:ti,ab OR belorussian:ti,ab OR belorussia:ti,ab OR belize:ti,ab OR bhutan:ti,ab OR bolivia:ti,ab OR bosnia:ti,ab OR herzegovina:ti,ab OR hercegovina:ti,ab OR botswana:ti,ab OR brasil:ti,ab OR brazil:ti,ab OR bulgaria:ti,ab OR 'burkina faso':ti,ab OR 'burkina fasso':ti,ab OR 'upper volta':ti,ab OR burundi:ti,ab OR urundi:ti,ab OR cambodia:ti,ab OR 'khmer republic':ti,ab OR kampuchea:ti,ab OR cameroon:ti,ab OR cameroons:ti,ab OR cameron:ti,ab OR 'cape verde':ti,ab OR 'central african republic':ti,ab OR chad:ti,ab OR china:ti,ab OR colombia:ti,ab OR comoros:ti,ab OR 'comoro islands':ti,ab OR comores:ti,ab OR mayotte:ti,ab OR congo:ti,ab OR zaire:ti,ab OR 'costa rica':ti,ab OR 'cote divoire':ti,ab OR 'ivory coast':ti,ab OR cuba:ti,ab OR djibouti:ti,ab OR 'french somaliland':ti,ab OR dominica:ti,ab OR 'dominican republic':ti,ab OR 'east timor':ti,ab OR 'east timur':ti,ab OR 'timor leste':ti,ab OR ecuador:ti,ab OR egypt:ti,ab OR 'el salvador':ti,ab OR eritrea:ti,ab OR ethiopia:ti,ab OR fiji:ti,ab OR gabon:ti,ab OR 'gabonese republic':ti,ab OR gambia:ti,ab OR gaza:ti,ab OR georgia:ti,ab OR ghana:ti,ab OR 'gold coast':ti,ab OR grenada:ti,ab OR guatemala:ti,ab OR guinea:ti,ab OR guam:ti,ab OR guiana:ti,ab OR guyana:ti,ab OR haiti:ti,ab OR honduras:ti,ab OR india:ti,ab OR indonesia:ti,ab OR iran:ti,ab OR iraq:ti,ab OR jamaica:ti,ab OR jordan:ti,ab OR kazakhstan:ti,ab OR kenya:ti,ab OR kiribati:ti,ab OR kosovo:ti,ab OR kyrgyzstan:ti,ab OR kirghizia:ti,ab OR 'kyrgyz republic':ti,ab OR kirghiz:ti,ab OR kirgizstan:ti,ab OR 'lao pdr':ti,ab OR laos:ti,ab OR latvia:ti,ab OR lebanon:ti,ab OR lesotho:ti,ab OR liberia:ti,ab OR libya:ti,ab OR macedonia:ti,ab OR madagascar:ti,ab OR malaysia:ti,ab OR malaya:ti,ab OR malay:ti,ab OR maldives:ti,ab OR malawi:ti,ab OR mali:ti,ab OR 'marshall islands':ti,ab OR mauritania:ti,ab OR mauritius:ti,ab OR mexico:ti,ab OR micronesia:ti,ab OR moldova:ti,ab OR moldovia:ti,ab OR mongolia:ti,ab OR morocco:ti,ab OR mozambique:ti,ab OR myanmar:ti,ab OR myanma:ti,ab OR burma:ti,ab OR namibia:ti,ab OR nepal:ti,ab OR nicaragua:ti,ab OR niger:ti,ab OR nigeria:ti,ab OR pakistan:ti,ab OR panama:ti,ab OR paraguay:ti,ab OR peru:ti,ab OR philippines:ti,ab OR philipines:ti,ab OR phillipines:ti,ab OR romania:ti,ab OR rumania:ti,ab OR roumania:ti,ab OR russian:ti,ab OR rwanda:ti,ab OR ruanda:ti,ab OR 'saint lucia':ti,ab OR 'st lucia':ti,ab OR 'saint vincent':ti,ab OR 'st vincent':ti,ab OR grenadines:ti,ab OR samoa:ti,ab OR 'sao tome':ti,ab OR senegal:ti,ab OR serbia:ti,ab OR montenegro:ti,ab OR 'sierra leone':ti,ab OR 'sri lanka':ti,ab OR ceylon:ti,ab OR 'solomon islands':ti,ab OR somalia:ti,ab OR sudan:ti,ab OR suriname:ti,ab OR surinam:ti,ab OR swaziland:ti,ab OR eswatini:ti,ab OR 'south africa' OR syria:ti,ab OR tajikistan:ti,ab OR tadzhikistan:ti,ab OR tadjikistan:ti,ab OR tanzania:ti,ab OR thailand:ti,ab OR togo:ti,ab OR tonga:ti,ab OR tunisia:ti,ab OR turkey:ti,ab OR turkmenistan:ti,ab OR tuvalu:ti,ab OR uganda:ti,ab OR ukraine:ti,ab OR russia:ti,ab OR ussr:ti,ab OR 'soviet union':ti,ab OR uzbekistan:ti,ab OR uzbek OR vanuatu:ti,ab OR venezuela:ti,ab OR

						vietnam:ti,ab OR 'viet nam':ti,ab OR 'west bank':ti,ab OR yemen:ti,ab OR zambia:ti,ab OR zimbabwe:ti,ab OR 'developing countries'/de OR 'africa'/de OR 'northern africa'/de OR 'africa south of the sahara'/de OR 'central africa'/de OR 'eastern africa'/de OR 'southern africa'/de OR 'western africa'/de OR 'asia'/de OR 'central asia'/de OR 'southeastern asia'/de OR 'western asia'/de OR 'caribbean'/de OR 'west indies'/de OR 'south america'/de OR 'latin america'/de OR 'central america'/de OR 'atlantic islands'/de OR 'pacific islands'/de OR 'indian ocean islands'/de OR 'afghanistan'/de OR 'albania'/de OR 'algeria'/de OR 'american samoa'/de OR 'angola'/de OR 'argentina'/de OR 'armenia'/de OR 'azerbaijan'/de OR 'bangladesh'/de OR 'barbados'/de OR 'benin'/de OR 'republic of belarus'/de OR 'belize'/de OR 'bhutan'/de OR 'bolivia'/de OR 'bosnia-herzegovina'/de OR 'botswana'/de OR 'brazil'/de OR 'bulgaria'/de OR 'burkina faso'/de OR 'burundi'/de OR 'cambodia'/de OR 'cameroon'/de OR 'cape verde'/de OR 'central african republic'/de OR 'chad'/de OR 'china'/de OR 'colombia'/de OR 'comoros'/de OR 'congo'/de OR 'costa rica'/de OR 'cote divoire' OR 'croatia'/de OR 'cuba'/de OR 'slovakia'/de OR 'djibouti'/de OR 'democratic republic of the congo'/de OR 'dominica'/de OR 'dominican republic'/de OR 'east timor'/de OR 'ecuador'/de OR 'egypt'/de OR 'el salvador'/de OR 'eritrea'/de OR 'ethiopia'/de OR 'equatorial guinea'/de OR 'fiji'/de OR 'french guiana'/de OR 'gabon'/de OR 'gambia'/de OR 'ghana'/de OR 'greece'/de OR 'grenada'/de OR 'guatemala'/de OR 'guinea'/de OR 'guinea-bissau'/de OR 'guam'/de OR 'guyana'/de OR 'haiti'/de OR 'honduras'/de OR 'independent state of samoa'/de OR 'india'/de OR 'indonesia'/de OR 'iran'/de OR 'iraq'/de OR 'jamaica'/de OR 'jordan'/de OR 'kazakhstan'/de OR 'kenya'/de OR 'korea'/de OR 'kyrgyzstan'/de OR 'laos'/de OR 'latvia'/de OR 'lebanon'/de OR 'lesotho'/de OR 'liberia'/de OR 'libya'/de OR 'macedonia' OR 'madagascar'/de OR 'malawi'/de OR 'malaysia'/de OR 'mali'/de OR 'malta'/de OR 'mauritania'/de OR 'mauritius'/de OR 'melanesia'/de OR 'mexico'/de OR 'micronesia'/de OR 'moldova'/de OR 'mongolia'/de OR 'morocco'/de OR 'mozambique'/de OR 'myanmar'/de OR 'namibia'/de OR 'nepal'/de OR 'nicaragua'/de OR 'niger'/de OR 'nigeria'/de OR 'pakistan'/de OR 'panama'/de OR 'papua new guinea'/de OR 'paraguay'/de OR 'peru'/de OR 'philippines'/de OR 'republic of korea'/de OR 'romania'/de OR 'russia'/de OR 'rwanda'/de OR 'saint lucia'/de OR 'saint vincent and the grenadines'/de OR 'samoa'/de OR 'senegal'/de OR 'serbia'/de OR 'montenegro'/de OR 'sierra leone'/de OR 'slovenia'/de OR 'sri lanka'/de OR 'somalia'/de OR 'south africa'/de OR 'sudan'/de OR 'suriname'/de OR 'swaziland'/de OR 'syria'/de OR 'tajikistan'/de OR 'tanzania'/de OR 'thailand'/de OR 'togo'/de OR 'tonga'/de OR 'tunisia'/de OR 'turkey'/de OR 'turkmenistan'/de OR 'uganda'/de OR 'ukraine'/de OR 'uruguay'/de OR 'ussr'/de OR 'uzbekistan'/de OR 'vanuatu'/de OR 'venezuela'/de OR 'vietnam'/de OR 'yemen'/de OR 'yugoslavia'/de OR 'zambia'/de OR 'zimbabwe'/de OR 'sub saharan africa' OR 'subsaharan africa')
**Combined search syntax**						
(‘hypertension’/de OR hypertension:ti,ab OR hypertensive:ti,ab OR ‘high blood pressure’:ti,ab OR ‘blood pressure’ OR diabetes:ti,ab OR diabet*:ti,ab OR dm2:ti,ab OR niddm:ti,ab OR dm 2:ti,ab OR t2d:ti,ab OR ‘dm type 2’:ti,ab OR ‘dm type II’:ti,ab OR dm1:ti,ab OR iddm:ti,ab OR dm 1:ti,ab OR t1d:ti,ab OR ‘dm type 1’:ti,ab OR ‘dm type I’:ti,ab OR ‘Diabetes Mellitus’/de) AND (‘support group*’:ti,ab OR buddy:ti,ab OR ‘self-help group*’:ti,ab OR ‘peer group*’:ti,ab OR ‘informal group*’:ti,ab OR ‘social group’:ti,ab OR ‘Volunteers’/de OR ‘Self-help groups’/de OR ‘Peer group’/de OR 'peer microfinance':ti,ab OR 'group medical visits':ti,ab) AND (financ*:ti,ab OR cash:ti,ab OR fund:ti,ab OR contribution:ti,ab OR support:ti,ab OR backing:ti,ab OR aid:ti,ab OR microfinance:ti,ab) AND ('emerging country' OR 'emerging countries' OR 'emerging nation' OR 'emerging nations' OR 'emerging population' OR 'emerging populations' OR 'developing country':ti,ab OR 'developing countries':ti,ab OR 'developing nation':ti,ab OR 'developing nations':ti,ab OR 'developing population':ti,ab OR 'developing populations':ti,ab OR 'developing world':ti,ab OR 'less developed country':ti,ab OR 'less developed countries':ti,ab OR 'less developed nation':ti,ab OR 'less developed nations':ti,ab OR 'less developed world':ti,ab OR 'lesser developed countries':ti,ab OR 'lesser developed nations':ti,ab OR 'under developed country':ti,ab OR 'under developed countries':ti,ab OR 'under developed nations':ti,ab OR 'under developed world':ti,ab OR 'underdeveloped country':ti,ab OR 'underdeveloped countries':ti,ab OR 'underdeveloped nation':ti,ab OR 'underdeveloped nations':ti,ab OR 'underdeveloped population':ti,ab OR 'underdeveloped populations':ti,ab OR 'underdeveloped world':ti,ab OR 'middle income country':ti,ab OR 'middle income countries':ti,ab OR 'middle income nation':ti,ab OR 'middle income nations':ti,ab OR 'middle income population':ti,ab OR 'middle income populations':ti,ab OR 'low income country':ti,ab OR 'low income countries':ti,ab OR 'low income nation':ti,ab OR 'low income nations':ti,ab OR 'low income population':ti,ab OR 'low income populations':ti,ab OR 'lower income country':ti,ab OR 'lower income countries':ti,ab OR 'lower income nations':ti,ab OR 'lower income population':ti,ab OR 'lower income populations':ti,ab OR 'underserved countries':ti,ab OR 'underserved nations':ti,ab OR 'underserved population':ti,ab OR 'underserved populations':ti,ab OR 'under served population':ti,ab OR 'under served populations':ti,ab OR 'deprived countries':ti,ab OR 'deprived population':ti,ab OR 'deprived populations':ti,ab OR 'poor country':ti,ab OR 'poor countries':ti,ab OR 'poor						

nation':ti,ab OR 'poor nations':ti,ab OR 'poor population':ti,ab OR 'poor populations':ti,ab OR 'poor world':ti,ab OR 'poorer countries':ti,ab OR 'poorer nations':ti,ab OR 'poorer population':ti,ab OR 'poorer populations':ti,ab OR 'developing economy':ti,ab OR 'developing economies':ti,ab OR 'less developed economy':ti,ab OR 'less developed economies':ti,ab OR 'underdeveloped economies':ti,ab OR 'middle income economy':ti,ab OR 'middle income economies':ti,ab OR 'low income economy':ti,ab OR 'low income economies':ti,ab OR 'lower income economies':ti,ab OR 'low gdp':ti,ab OR 'low gnp':ti,ab OR 'low gross domestic':ti,ab OR 'low gross national':ti,ab OR 'lower gdp':ti,ab OR 'lower gross domestic':ti,ab OR lmic:ti,ab OR lmics:ti,ab OR 'third world':ti,ab OR 'lami country':ti,ab OR 'lami countries':ti,ab OR 'transitional country':ti,ab OR 'transitional countries':ti,ab OR africa:ti,ab OR asia:ti,ab OR caribbean:ti,ab OR 'west indies':ti,ab OR 'south america':ti,ab OR 'latin america':ti,ab OR 'central america':ti,ab OR 'atlantic islands':ti,ab OR 'pacific islands':ti,ab OR 'indian ocean islands':ti,ab OR afghanistan:ti,ab OR albania:ti,ab OR algeria:ti,ab OR angola:ti,ab OR argentina:ti,ab OR armenia:ti,ab OR azerbaijan:ti,ab OR bangladesh:ti,ab OR barbados:ti,ab OR benin:ti,ab OR byelarus:ti,ab OR byelorussian:ti,ab OR belarus:ti,ab OR belorussian:ti,ab OR belorussia:ti,ab OR belize:ti,ab OR bhutan:ti,ab OR bolivia:ti,ab OR bosnia:ti,ab OR herzegovina:ti,ab OR hercegovina:ti,ab OR botswana:ti,ab OR brasil:ti,ab OR brazil:ti,ab OR bulgaria:ti,ab OR 'burkina faso':ti,ab OR 'burkina fasso':ti,ab OR 'upper volta':ti,ab OR burundi:ti,ab OR urundi:ti,ab OR cambodia:ti,ab OR 'khmer republic':ti,ab OR kampuchea:ti,ab OR cameroon:ti,ab OR cameroons:ti,ab OR cameron:ti,ab OR 'cape verde':ti,ab OR 'central african republic':ti,ab OR chad:ti,ab OR china:ti,ab OR colombia:ti,ab OR comoros:ti,ab OR 'comoro islands':ti,ab OR comores:ti,ab OR mayotte:ti,ab OR congo:ti,ab OR zaire:ti,ab OR 'costa rica':ti,ab OR 'cote divoire':ti,ab OR 'ivory coast':ti,ab OR cuba:ti,ab OR djibouti:ti,ab OR 'french somaliland':ti,ab OR dominica:ti,ab OR 'dominican republic':ti,ab OR 'east timor':ti,ab OR 'east timur':ti,ab OR 'timor leste':ti,ab OR ecuador:ti,ab OR egypt:ti,ab OR 'el salvador':ti,ab OR eritrea:ti,ab OR ethiopia:ti,ab OR fiji:ti,ab OR gabon:ti,ab OR 'gabonese republic':ti,ab OR gambia:ti,ab OR gaza:ti,ab OR georgia:ti,ab OR ghana:ti,ab OR 'gold coast':ti,ab OR grenada:ti,ab OR guatemala:ti,ab OR guinea:ti,ab OR guam:ti,ab OR guiana:ti,ab OR guyana:ti,ab OR haiti:ti,ab OR honduras:ti,ab OR india:ti,ab OR indonesia:ti,ab OR iran:ti,ab OR iraq:ti,ab OR jamaica:ti,ab OR jordan:ti,ab OR kazakhstan:ti,ab OR kenya:ti,ab OR kiribati:ti,ab OR kosovo:ti,ab OR kyrgyzstan:ti,ab OR kirghizia:ti,ab OR 'kyrgyz republic':ti,ab OR kirghiz:ti,ab OR kirgizstan:ti,ab OR 'lao pdr':ti,ab OR laos:ti,ab OR latvia:ti,ab OR lebanon:ti,ab OR lesotho:ti,ab OR liberia:ti,ab OR libya:ti,ab OR macedonia:ti,ab OR madagascar:ti,ab OR malaysia:ti,ab OR malaya:ti,ab OR malay:ti,ab OR maldives:ti,ab OR malawi:ti,ab OR mali:ti,ab OR 'marshall islands':ti,ab OR mauritania:ti,ab OR mauritius:ti,ab OR mexico:ti,ab OR micronesia:ti,ab OR moldova:ti,ab OR moldovia:ti,ab OR mongolia:ti,ab OR morocco:ti,ab OR mozambique:ti,ab OR myanmar:ti,ab OR myanma:ti,ab OR burma:ti,ab OR namibia:ti,ab OR nepal:ti,ab OR nicaragua:ti,ab OR niger:ti,ab OR nigeria:ti,ab OR pakistan:ti,ab OR panama:ti,ab OR paraguay:ti,ab OR peru:ti,ab OR philippines:ti,ab OR philipines:ti,ab OR phillipines:ti,ab OR romania:ti,ab OR rumania:ti,ab OR roumania:ti,ab OR russian:ti,ab OR rwanda:ti,ab OR ruanda:ti,ab OR 'saint lucia':ti,ab OR 'st lucia':ti,ab OR 'saint vincent':ti,ab OR 'st vincent':ti,ab OR grenadines:ti,ab OR samoa:ti,ab OR 'sao tome':ti,ab OR senegal:ti,ab OR serbia:ti,ab OR montenegro:ti,ab OR 'sierra leone':ti,ab OR 'sri lanka':ti,ab OR ceylon:ti,ab OR 'solomon islands':ti,ab OR somalia:ti,ab OR sudan:ti,ab OR suriname:ti,ab OR surinam:ti,ab OR swaziland:ti,ab OR eswatini:ti,ab OR 'south africa' OR syria:ti,ab OR tajikistan:ti,ab OR tadzhikistan:ti,ab OR tadjikistan:ti,ab OR tanzania:ti,ab OR thailand:ti,ab OR togo:ti,ab OR tonga:ti,ab OR tunisia:ti,ab OR turkey:ti,ab OR turkmenistan:ti,ab OR tuvalu:ti,ab OR uganda:ti,ab OR ukraine:ti,ab OR russia:ti,ab OR ussr:ti,ab OR 'soviet union':ti,ab OR uzbekistan:ti,ab OR uzbek OR vanuatu:ti,ab OR venezuela:ti,ab OR vietnam:ti,ab OR 'viet nam':ti,ab OR 'west bank':ti,ab OR yemen:ti,ab OR zambia:ti,ab OR zimbabwe:ti,ab OR 'developing countries'/de OR 'africa'/de OR 'northern africa'/de OR 'africa south of the sahara'/de OR 'central africa'/de OR 'eastern africa'/de OR 'southern africa'/de OR 'western africa'/de OR 'asia'/de OR 'central asia'/de OR 'southeastern asia'/de OR 'western asia'/de OR 'caribbean'/de OR 'west indies'/de OR 'south america'/de OR 'latin america'/de OR 'central america'/de OR 'atlantic islands'/de OR 'pacific islands'/de OR 'indian ocean islands'/de OR 'afghanistan'/de OR 'albania'/de OR 'algeria'/de OR 'american samoa'/de OR 'angola'/de OR 'argentina'/de OR 'armenia'/de OR 'azerbaijan'/de OR 'bangladesh'/de OR 'barbados'/de OR 'benin'/de OR 'republic of belarus'/de OR 'belize'/de OR 'bhutan'/de OR 'bolivia'/de OR 'bosnia-herzegovina'/de OR 'botswana'/de OR 'brazil'/de OR 'bulgaria'/de OR 'burkina faso'/de OR 'burundi'/de OR 'cambodia'/de OR 'cameroon'/de OR 'cape verde'/de OR 'central african republic'/de OR 'chad'/de OR 'china'/de OR 'colombia'/de OR 'comoros'/de OR 'congo'/de OR 'costa rica'/de OR 'cote divoire' OR 'croatia'/de OR 'cuba'/de OR 'slovakia'/de OR 'djibouti'/de OR 'democratic republic of the congo'/de OR 'dominica'/de OR 'dominican republic'/de OR 'east timor'/de OR 'ecuador'/de OR 'egypt'/de OR 'el salvador'/de OR 'eritrea'/de OR 'ethiopia'/de OR 'equatorial guinea'/de OR 'fiji'/de OR 'french guiana'/de OR 'gabon'/de OR 'gambia'/de OR 'ghana'/de OR 'greece'/de OR 'grenada'/de OR 'guatemala'/de OR 'guinea'/de OR 'guinea-bissau'/de OR 'guam'/de OR 'guyana'/de OR 'haiti'/de OR 'honduras'/de OR 'independent state of samoa'/de OR 'india'/de OR 'indonesia'/de OR 'iran'/de OR 'iraq'/de OR 'jamaica'/de OR 'jordan'/de OR 'kazakhstan'/de OR 'kenya'/de OR 'korea'/de OR 'kyrgyzstan'/de OR 'laos'/de OR 'latvia'/de OR 'lebanon'/de OR 'lesotho'/de OR 'liberia'/de OR 'libya'/de OR 'macedonia' OR 'madagascar'/de OR 'malawi'/de OR 'malaysia'/de OR 'mali'/de OR 'malta'/de OR 'mauritania'/de OR 'mauritius'/de OR 'melanesia'/de OR 'mexico'/de OR 'micronesia'/de OR 'moldova'/de OR 'mongolia'/de OR 'morocco'/de OR 'mozambique'/de OR 'myanmar'/de OR 'namibia'/de OR 'nepal'/de OR 'nicaragua'/de OR 'niger'/de OR 'nigeria'/de OR 'pakistan'/de OR 'panama'/de OR 'papua new guinea'/de OR 'paraguay'/de OR 'peru'/de OR 'philippines'/de OR 'republic of korea'/de OR 'romania'/de OR 'russia'/de OR 'rwanda'/de OR 'saint lucia'/de OR 'saint vincent and the grenadines'/de OR 'samoa'/de OR 'senegal'/de OR 'serbia'/de OR 'montenegro'/de OR 'sierra leone'/de OR 'slovenia'/de OR 'sri lanka'/de OR 'somalia'/de OR 'south africa'/de OR 'sudan'/de OR 'suriname'/de OR 'swaziland'/de OR 'syria'/de OR 'tajikistan'/de OR 'tanzania'/de OR 'thailand'/de OR 'togo'/de OR 'tonga'/de OR 'tunisia'/de OR 'turkey'/de OR 'turkmenistan'/de OR 'uganda'/de OR 'ukraine'/de OR 'uruguay'/de OR 'ussr'/de OR 'uzbekistan'/de OR 'vanuatu'/de OR 'venezuela'/de OR 'vietnam'/de OR 'yemen'/de OR 'yugoslavia'/de OR 'zambia'/de OR 'zimbabwe'/de OR 'sub saharan africa' OR 'subsaharan africa')

**Scopus**						
Diabetes OR hypertension		Patient support groups		Financial component		Low and Middle Income Countries (LMICs)
(hypertens* OR “high blood pressure” OR “blood pressure” OR diabet* OR dm2 OR niddm OR dm 2 OR t2d	AND	(“support group*” OR buddy OR “self-help group*” OR “peer group*” OR “informal group*” OR “social	AND	(financ* OR cash OR fund OR contribution OR support OR	AND	(“emerging country” OR “emerging countries” OR “emerging nation” OR “emerging nations” OR “emerging population” OR “emerging populations” OR “developing country” OR “developing countries” OR “developing nation” OR “developing nations” OR “developing population” OR “developing populations” OR “developing world” OR “less developed country” OR “less developed countries” OR “less developed nation” OR “less developed nations” OR “less developed world” OR “lesser developed countries” OR “lesser developed

OR “dm type 2” OR “dm type II” OR dm1 OR iddm OR “dm 1” OR t1d OR “dm type 1” OR “dm type I” OR "Diabetes Mellitus")		group” OR volunteer* OR “Self-help groups" OR “peer microfinance“ OR “group medical visits“)		backing OR aid OR microfinance)		nations” OR “under developed country” OR “under developed countries” OR “under developed nations” OR “under developed world” OR “underdeveloped country” OR “underdeveloped countries” OR “underdeveloped nation” OR “underdeveloped nations” OR “underdeveloped population” OR “underdeveloped populations” OR “underdeveloped world” OR “middle income country” OR “middle income countries” OR “middle income nation” OR “middle income nations” OR “middle income population” OR “middle income populations” OR “low income country” OR “low income countries” OR “low income nation” OR “low income nations” OR “low income population” OR “low income populations” OR “lower income country” OR “lower income countries” OR “lower income nations” OR “lower income population” OR “lower income populations” OR “underserved countries” OR “underserved nations” OR “underserved population” OR “underserved populations” OR “under served population” OR “under served populations” OR “deprived countries” OR “deprived population” OR “deprived populations” OR “poor country” OR “poor countries” OR “poor nation” OR “poor nations” OR “poor population” OR “poor populations” OR “poor world” OR “poorer countries” OR “poorer nations” OR “poorer population” OR “poorer populations” OR “developing economy” OR “developing economies” OR “less developed economy” OR “less developed economies” OR “underdeveloped economies” OR “middle income economy” OR “middle income economies” OR “low income economy” OR “low income economies” OR “lower income economies” OR “low gdp” OR “low gnp” OR “low gross domestic” OR “low gross national” OR “lower gdp” OR “lower gross domestic” OR lmic OR lmics OR “third world” OR “lami country” OR “lami countries” OR “transitional country” OR “transitional countries” OR Africa OR Asia OR Caribbean OR “West Indies” OR “South America” OR “Latin America” OR “Central America” OR “Atlantic Islands” OR “Pacific Islands” OR “Indian Ocean Islands” OR Afghanistan OR Albania OR Algeria OR Angola OR Argentina OR Armenia OR Azerbaijan OR Bangladesh OR Barbados OR Benin OR Byelarus OR Byelorussian OR Belarus OR Belorussian OR Belorussia OR Belize OR Bhutan OR Bolivia OR Bosnia OR Herzegovina OR Hercegovina OR Botswana OR Brasil OR Brazil OR Bulgaria OR “Burkina Faso” OR “Burkina Fasso” OR “Upper Volta” OR Burundi OR Urundi OR Cambodia OR “Khmer Republic” OR Kampuchea OR Cameroon OR Cameroons OR Cameron OR Cape Verde OR “Central African Republic” OR Chad OR China OR Colombia OR Comoros OR Comoro Islands OR Comores OR Mayotte OR Congo OR Zaire OR Costa Rica OR “Cote d'Ivoire” OR “Ivory Coast” OR Cuba OR Djibouti OR “French Somaliland” OR Dominica OR “Dominican Republic” OR “East Timor” OR “East Timur” OR “Timor Leste” OR Ecuador OR Egypt OR “El Salvador” OR Eritrea OR Ethiopia OR Fiji OR Gabon OR “Gabonese Republic” OR Gambia OR Gaza OR Georgia OR Ghana OR Gold Coast OR Grenada OR Guatemala OR Guinea OR Guam OR Guiana OR Guyana OR Haiti OR Honduras OR India OR Indonesia OR Iran OR Iraq OR Jamaica OR Jordan OR Kazakhstan OR Kenya OR Kiribati OR Kosovo OR Kyrgyzstan OR Kirghizia OR “Kyrgyz Republic” OR Kirghiz OR Kirgizstan OR “Lao PDR” OR Laos OR Latvia OR Lebanon OR Lesotho OR Liberia OR Libya OR Macedonia OR Madagascar OR Malaysia OR Malaya OR Malay OR Maldives OR Malawi OR Mali OR Marshall Islands OR Mauritania OR Mauritius OR Mexico OR Micronesia OR Moldova OR Moldovia OR Mongolia OR Montenegro OR Morocco OR Mozambique OR Myanmar OR Myanma OR Burma OR Namibia OR Nepal OR Nicaragua OR Niger OR Nigeria OR Pakistan OR Panama OR Paraguay OR Peru OR Philippines OR Philipines OR Phillipines OR Romania OR Rumania OR Roumania OR Russia OR Russian OR Rwanda OR Ruanda OR “Saint Lucia” OR “St Lucia” OR “Saint Vincent” OR “St Vincent” OR Grenadines OR Samoa OR Sao Tome OR Senegal OR Serbia OR Montenegro OR “Sierra Leone” OR “Sri Lanka” OR Ceylon OR “Solomon Islands” OR Somalia OR Sudan OR Suriname OR Surinam OR Swaziland OR Eswatini OR “South Africa” OR Syria OR Tajikistan OR Tadzhikistan OR Tadjikistan OR Tanzania OR Thailand OR Togo OR Tonga OR Tunisia OR Turkey OR Turkmenistan OR Tuvalu OR Uganda OR Ukraine OR Russia OR USSR OR “Soviet Union” OR Uzbekistan OR Uzbek OR Vanuatu OR Venezuela OR Vietnam OR “Viet Nam” OR “West Bank” OR Yemen OR Zambia OR Zimbabwe OR “Sub Saharan Africa” OR “SubSaharan Africa”)

**Combined search syntax**
(hypertens* OR "high blood pressure" OR "blood pressure" OR diabet* OR dm2 OR niddm OR dm 2 OR t2d OR "dm type 2" OR "dm type II" OR dm1 OR iddm OR "dm 1" OR t1d OR "dm type 1" OR "dm type I" OR "Diabetes Mellitus") AND ("support group*" OR buddy OR "self-help group*" OR "peer group*" OR "informal group*" OR "social group" OR volunteer* OR "Self-help groups" OR "peer microfinance" OR "group medical visits") AND (financ* OR cash OR fund OR contribution OR support OR backing OR aid OR microfinance) AND ("emerging country" OR "emerging countries" OR "emerging nation" OR "emerging nations" OR "emerging population" OR "emerging populations" OR "developing country" OR "developing countries" OR "developing nation" OR "developing nations" OR "developing population" OR "developing populations" OR "developing world" OR "less developed country" OR "less developed countries" OR "less developed nation" OR "less developed nations" OR "less developed world" OR "lesser developed countries" OR "lesser developed nations" OR "under developed country" OR "under developed countries" OR "under developed nations" OR "under developed world" OR "underdeveloped country" OR "underdeveloped countries" OR "underdeveloped nation" OR "underdeveloped nations" OR "underdeveloped population" OR "underdeveloped populations" OR "underdeveloped world" OR "middle income country" OR "middle income countries" OR "middle income nation" OR "middle income nations" OR "middle income population" OR "middle income populations" OR "low income country" OR "low income countries" OR "low income nation" OR "low income nations" OR "low income population" OR "low income populations" OR "lower income country" OR "lower income countries" OR "lower income nations" OR "lower income population" OR "lower income populations" OR "underserved countries" OR "underserved nations" OR "underserved population" OR "underserved populations" OR "under served population" OR "under served populations" OR "deprived countries" OR "deprived population" OR "deprived populations" OR "poor country" OR "poor countries" OR "poor nation" OR "poor nations" OR "poor population" OR "poor populations" OR "poor world" OR "poorer countries" OR "poorer nations" OR "poorer population" OR "poorer populations" OR "developing economy" OR "developing economies" OR "less developed economy" OR "less developed economies" OR "underdeveloped economies" OR "middle income economy" OR "middle income economies" OR "low income economy" OR "low income economies" OR "lower income economies" OR "low gdp" OR "low gnp" OR "low gross domestic" OR "low gross national" OR "lower gdp" OR "lower gross domestic" OR lmic OR lmics OR "third world" OR "lami country" OR "lami countries" OR "transitional country" OR "transitional countries" OR Africa OR Asia OR Caribbean OR "West Indies" OR "South America" OR "Latin America" OR "Central America" OR "Atlantic Islands" OR "Pacific Islands" OR "Indian Ocean Islands")

Web of Science

Diabetes OR hypertension		Patient support groups		Financial component		Low and Middle Income Countries (LMICs)
(hypertension OR hypertensive OR "high blood pressure" OR "blood pressure" OR diabetes OR diabetic OR dm2 OR niddm OR "dm 2" OR t2d OR "dm type 2" OR "dm type II" OR dm1 OR iddm OR "dm 1" OR t1d OR "dm type 1" OR "dm type I" OR "Diabetes Mellitus")	AND	("support group*"OR buddy OR "self-help group*" OR "peer group*" OR "informal group*" OR "social group" OR "Volunteers" OR "Self-help groups" OR "peer/microfinance" OR "group medical visits")	AND	(financ* OR cash OR fund OR contribution OR support OR backing OR aid OR microfinance)	AND	("emerging countries" OR "emerging nations" OR "emerging populations" OR "developing countries" OR "developing nations" OR "developing populations" OR "developing world" OR "less developed countries" OR "less developed nations" OR "less developed world" OR "lesser developed countries" OR "lesser developed nations" OR "under developed countries" OR "under developed nations" OR "under developed world" OR "underdeveloped countries" OR "underdeveloped nations" OR "underdeveloped populations" OR "underdeveloped world" OR "middle income countries" OR "middle income nations" OR "middle income populations" OR "low income countries" OR "low income nations" OR "low income populations" OR "lower income countries" OR "lower income nations" OR "lower income populations" OR "underserved countries" OR "underserved nations" OR "underserved populations" OR "under served population" OR "deprived countries" OR "deprived populations" OR "poor countries" OR "poor nations" OR "poor populations" OR "poor world" OR "poorer countries" OR "poorer nations" OR "poorer populations" OR "developing economies" OR "less developed economies" OR "middle income economy" OR "middle income economies" OR "low income economies" OR "lower income economies" OR "low gdp" OR "low gnp" OR "low gross domestic" OR "low gross national" OR "lower gdp" OR "lower gross domestic" OR lmic OR lmics OR "third world" OR "lami country" OR "lami countries" OR "transitional country" OR "transitional countries" OR Africa OR Asia OR Caribbean OR "West Indies" OR "South America" OR "Latin America"OR "Central America" OR "Atlantic Islands" OR "Pacific Islands" OR "Indian Ocean Islands" OR Afghanistan OR Albania OR Algeria OR Angola OR Argentina OR Armenia OR Azerbaijan OR Bangladesh OR Barbados OR Benin OR Byelarus OR Byelorussian OR Belarus OR Belorussian OR Belorussia OR Belize OR Bhutan OR Bolivia OR Bosnia OR Herzegovina OR Hercegovina OR Botswana OR Brasil OR Brazil OR Bulgaria OR "Burkina Faso" OR "Burkina Fasso" OR "Upper Volta" OR Burundi OR Urundi OR Cambodia OR "Khmer Republic" OR Kampuchea OR Cameroon OR Cameroons OR Cameron OR Cape Verde OR Central African Republic OR Chad OR China OR Colombia OR Comoros OR "Comoro Islands" OR Comores OR Mayotte OR Congo OR Zaire OR Costa Rica OR {Cote d'Ivoire} OR {Ivory Coast }OR Cuba OR Djibouti OR French Somaliland OR Dominica OR {Dominican Republic} OR {East Timor} OR {East Timur} OR {Timor Leste} OR Ecuador OR Egypt OR {El Salvador} OR Eritrea

						OR Ethiopia OR Fiji OR Gabon OR {Gabonese Republic} OR Gambia OR Gaza OR Georgia OR Ghana OR {Gold Coast} OR Grenada OR Guatemala OR Guinea OR Guam OR Guiana OR Guyana OR Haiti OR Honduras OR India OR Indonesia OR Iran OR Iraq OR Jamaica OR Jordan OR Kazakhstan OR Kenya OR Kiribati OR Kosovo OR Kyrgyzstan OR Kirghizia OR Kyrgyz Republic OR Kirghiz OR Kirgizstan OR "Lao PDR" OR Laos OR Latvia OR Lebanon OR Lesotho OR Liberia OR Libya OR Macedonia OR Madagascar OR Malaysia OR Malaya OR Malay OR Maldives OR Malawi OR Mali OR Marshall Islands OR Mauritania OR Mauritius OR Mexico OR Micronesia OR Moldova OR Moldovia OR Mongolia OR Montenegro OR Morocco OR Mozambique OR Myanmar OR Myanma OR Burma OR Namibia OR Nepal OR Nicaragua OR Niger OR Nigeria OR Pakistan OR Panama OR Paraguay OR Peru OR Philippines OR Philipines OR Phillipines OR Romania OR Rumania OR Roumania OR Russia OR Russian OR Rwanda OR Ruanda OR Saint Lucia OR St Lucia OR Saint Vincent OR St Vincent OR Grenadines OR Samoa OR Sao Tome OR Senegal OR Serbia OR Montenegro OR Sierra Leone OR Sri Lanka OR Ceylon OR {Solomon Islands} OR Somalia OR Sudan OR Suriname OR Surinam OR Swaziland OR Eswatini OR {South Africa} OR Syria OR Tajikistan OR Tadzhikistan OR Tadjikistan OR Tanzania OR Thailand OR Togo OR Tonga OR Tunisia OR Turkey OR Turkmenistan OR Tuvalu OR Uganda OR Ukraine OR Russia OR USSR OR {Soviet Union} OR Uzbekistan OR Uzbek OR Vanuatu OR Venezuela OR Vietnam OR Viet Nam OR {West Bank} OR Yemen OR Zambia OR Zimbabwe OR {Developing Countries} OR Africa OR North Africa OR “Africa South of the Sahara” OR “Central Africa” OR “East Africa” OR “South Africa” OR “West Africa” OR Asia OR Central Asia OR Asia, “Southeast Asia” OR “Western Asia” OR “Caribbean Region” OR West Indies OR “South America” OR {Latin America} OR {Central America} OR {Atlantic Islands} OR "Pacific Islands" OR "Indian Ocean Islands" OR “Sub Saharan Africa " OR “SubSaharan Africa ")
**Combined search syntax**						
(hypertension OR hypertensive OR “high blood pressure” OR “blood pressure” OR diabetes OR diabetic OR dm2 OR niddm OR "dm 2" OR t2d OR “dm type 2” OR “dm type II” OR dm1 OR iddm OR "dm 1" OR t1d OR “dm type 1” OR “dm type I” OR "Diabetes Mellitus") AND (“support group*”OR buddy OR “self-help group*” OR “peer group*” OR “informal group*” OR “social group” OR “Volunteers" OR “Self-help groups" OR "peer/microfinance" OR "group medical visits") AND (financ* OR cash OR fund OR contribution OR support OR backing OR aid OR microfinance) AND (“emerging countries” OR “emerging nations” OR “emerging populations” OR "developing countries" OR "developing nations" OR "developing populations" OR "developing world" OR "less developed countries" OR "less developed nations" OR "less developed world" OR "lesser developed countries" OR "lesser developed nations" OR "under developed countries" OR "under developed nations" OR "under developed world" OR "underdeveloped countries" OR "underdeveloped nations" OR "underdeveloped populations" OR "underdeveloped world" OR "middle income countries" OR "middle income nations" OR "middle income populations" OR "low income countries" OR "low income nations" OR "low income populations" OR "lower income countries" OR "lower income nations" OR "lower income populations" OR "underserved countries" OR "underserved nations" OR "underserved populations" OR "under served population" OR "deprived countries" OR "deprived populations" OR "poor countries" OR "poor nations" OR "poor populations" OR "poor world" OR "poorer countries" OR "poorer nations" OR "poorer populations" OR "developing economies" OR "less developed economies" OR "middle income economy" OR "middle income economies" OR "low income economies" OR "lower income economies" OR "low gdp" OR "low gnp" OR "low gross domestic" OR "low gross national" OR "lower gdp" OR "lower gross domestic" OR lmic OR lmics OR "third world" OR "lami country" OR "lami countries" OR "transitional country" OR "transitional countries" OR Africa OR Asia OR Caribbean OR “West Indies” OR “South America” OR “Latin America”OR “Central America” OR "Atlantic Islands" OR "Pacific Islands" OR "Indian Ocean Islands" OR Afghanistan OR Albania OR Algeria OR Angola OR Argentina OR Armenia OR Azerbaijan OR Bangladesh OR Barbados OR Benin OR Byelarus OR Byelorussian OR Belarus OR Belorussian OR Belorussia OR Belize OR Bhutan OR Bolivia OR Bosnia OR Herzegovina OR Hercegovina OR Botswana OR Brasil OR Brazil OR Bulgaria OR “Burkina Faso” OR “Burkina Fasso” OR “Upper Volta” OR Burundi OR Urundi OR Cambodia OR “Khmer Republic” OR Kampuchea OR Cameroon OR Cameroons OR Cameron OR Cape Verde OR Central African Republic OR Chad OR China OR Colombia OR Comoros OR “Comoro Islands” OR Comores OR Mayotte OR Congo OR Zaire OR Costa Rica OR {Cote d'Ivoire} OR {Ivory Coast }OR Cuba OR Djibouti OR French Somaliland OR Dominica OR {Dominican Republic} OR {East Timor} OR {East Timur} OR {Timor Leste} OR Ecuador OR Egypt OR {El Salvador} OR Eritrea OR Ethiopia OR Fiji OR Gabon OR {Gabonese Republic} OR Gambia OR Gaza OR Georgia OR Ghana OR {Gold Coast} OR Grenada OR Guatemala OR Guinea OR Guam OR Guiana OR Guyana OR Haiti OR Honduras OR India OR Indonesia OR Iran OR Iraq OR Jamaica OR Jordan OR Kazakhstan OR Kenya OR Kiribati OR Kosovo OR Kyrgyzstan OR Kirghizia OR Kyrgyz Republic OR Kirghiz OR Kirgizstan OR "Lao PDR" OR Laos OR Latvia OR Lebanon OR Lesotho OR Liberia OR Libya OR Macedonia OR Madagascar OR Malaysia OR Malaya OR Malay OR Maldives OR Malawi OR Mali OR Marshall Islands OR Mauritania OR Mauritius OR Mexico OR Micronesia OR Moldova OR Moldovia OR Mongolia OR Montenegro OR Morocco OR Mozambique OR Myanmar OR Myanma OR Burma OR Namibia OR Nepal OR Nicaragua OR Niger OR Nigeria OR Pakistan OR Panama OR Paraguay OR Peru OR Philippines OR Philipines OR Phillipines OR Romania OR Rumania OR Roumania OR Russia OR Russian OR Rwanda OR Ruanda OR Saint Lucia OR St Lucia OR Saint Vincent OR St Vincent OR Grenadines OR Samoa OR Sao Tome OR Senegal OR Serbia OR Montenegro OR Sierra Leone OR Sri Lanka OR Ceylon OR {Solomon Islands} OR Somalia OR Sudan OR Suriname OR Surinam OR Swaziland OR Eswatini OR {South Africa} OR Syria OR Tajikistan OR Tadzhikistan OR Tadjikistan OR Tanzania OR Thailand OR Togo OR Tonga OR Tunisia OR Turkey OR Turkmenistan OR Tuvalu OR Uganda OR Ukraine OR Russia OR USSR OR {Soviet Union} OR Uzbekistan OR Uzbek OR Vanuatu OR Venezuela OR Vietnam OR Viet Nam OR {West Bank} OR Yemen OR Zambia OR Zimbabwe OR {Developing Countries} OR Africa OR North Africa OR “Africa South of the Sahara” OR “Central Africa” OR “East Africa” OR “South Africa” OR						

“West Africa” OR Asia OR Central Asia OR Asia, “Southeast Asia” OR “Western Asia” OR “Caribbean Region” OR West Indies OR “South America” OR {Latin America} OR {Central America} OR {Atlantic Islands} OR "Pacific Islands" OR "Indian Ocean Islands" OR “Sub Saharan Africa " OR “SubSaharan Africa ")						
**Global Health**						
Diabetes OR hypertension		Patient support groups		Financial component		Low and Middle Income Countries (LMICs)
(hypertension OR hypertensive OR “high blood pressure” OR “blood pressure” OR diabetes OR diabet* OR dm2 OR niddm OR dm 2 OR t2d OR “dm type 2” OR “dm type II” OR dm1 OR iddm OR "dm 1" OR t1d OR “dm type 1” OR “dm type I” OR "Diabetes Mellitus")	AND	(“support group*” OR buddy OR “self-help group*” OR “peer group*” OR “informal group*” OR “social group” OR “Volunteers" OR “Self-help groups" OR “Peer group" OR "peer/microfinance" OR "group medical visits")	AND	(finance OR financing OR financial OR cash OR fund OR contribution OR support OR backing OR aid OR microfinance)	AND	(“emerging country” OR “emerging countries” OR “emerging nation” OR “emerging nations” OR “emerging population” OR “emerging populations” OR “developing country” OR “developing countries” OR “developing nation” OR “developing nations” OR “developing population” OR “developing populations” OR “developing world” OR “less developed country” OR “less developed countries” OR “less developed nation” OR “less developed nations” OR “less developed world” OR “lesser developed countries” OR “lesser developed nations” OR “under developed country” OR “under developed countries” OR “under developed nations” OR “under developed world” OR “underdeveloped country” OR “underdeveloped countries” OR “underdeveloped nation” OR “underdeveloped nations” OR “underdeveloped population” OR “underdeveloped populations” OR “underdeveloped world” OR “middle income country” OR “middle income countries” OR “middle income nation” OR “middle income nations” OR “middle income population” OR “middle income populations” OR “low income country” OR “low income countries” OR “low income nation” OR “low income nations” OR “low income population” OR “low income populations” OR “lower income country” OR “lower income countries” OR “lower income nations” OR “lower income population” OR “lower income populations” OR “underserved countries” OR “underserved nations” OR “underserved population” OR “underserved populations” OR “under served population” OR “under served populations” OR “deprived countries” OR “deprived population” OR “deprived populations” OR “poor country” OR “poor countries” OR “poor nation” OR “poor nations” OR “poor population” OR “poor populations” OR “poor world” OR “poorer countries” OR “poorer nations” OR “poorer population” OR “poorer populations” OR “developing economy” OR “developing economies” OR “less developed economy” OR “less developed economies” OR “underdeveloped economies” OR “middle income economy” OR “middle income economies” OR “low income economy” OR “low income economies” OR “lower income economies” OR “low gdp” OR “low gnp” OR “low gross domestic” OR “low gross national” OR “lower gdp” OR “lower gross domestic” OR lmic OR lmics OR “third world” OR “lami country” OR “lami countries” OR “transitional country” OR “transitional countries” OR Africa OR Asia OR Caribbean OR “West Indies” OR “South America” OR “Latin America” OR “Central America” OR “Atlantic Islands” OR “Pacific Islands” OR “Indian Ocean Islands” OR Afghanistan OR Albania OR Algeria OR Angola OR Argentina OR Armenia OR Azerbaijan OR Bangladesh OR Barbados OR Benin OR Byelarus OR Byelorussian OR Belarus OR Belorussian OR Belorussia OR Belize OR Bhutan OR Bolivia OR Bosnia OR Herzegovina OR Hercegovina OR Botswana OR Brasil OR Brazil OR Bulgaria OR “Burkina Faso” OR “Burkina Fasso” OR “Upper Volta” OR Burundi OR Urundi OR Cambodia OR “Khmer Republic” OR Kampuchea OR Cameroon OR Cameroons OR Cameron OR Cape Verde OR “Central African Republic” OR Chad OR China OR Colombia OR Comoros OR Comoro Islands OR Comores OR Mayotte OR Congo OR Zaire OR Costa Rica OR “Cote d'Ivoire” OR “Ivory Coast” OR Cuba OR Djibouti OR “French Somaliland” OR Dominica OR “Dominican Republic” OR “East Timor” OR “East Timur” OR “Timor Leste” OR Ecuador OR Egypt OR “El Salvador” OR Eritrea OR Ethiopia OR Fiji OR Gabon OR “Gabonese Republic” OR Gambia OR Gaza OR Georgia OR Ghana OR Gold Coast OR Grenada OR Guatemala OR Guinea OR Guam OR Guiana OR Guyana OR Haiti OR Honduras OR India OR Indonesia OR Iran OR Iraq OR Jamaica OR Jordan OR Kazakhstan OR Kenya OR Kiribati OR Kosovo OR Kyrgyzstan OR Kirghizia OR “Kyrgyz Republic” OR Kirghiz OR Kirgizstan OR “Lao PDR” OR Laos OR Latvia OR Lebanon OR Lesotho OR Liberia OR Libya OR Macedonia OR Madagascar OR Malaysia OR Malaya OR Malay OR Maldives OR Malawi OR Mali OR Marshall Islands OR Mauritania OR Mauritius OR Mexico OR Micronesia OR Moldova OR Moldovia OR Mongolia OR Montenegro

						OR Morocco OR Mozambique OR Myanmar OR Myanma OR Burma OR Namibia OR Nepal OR Nicaragua OR Niger OR Nigeria OR Pakistan OR Panama OR Paraguay OR Peru OR Philippines OR Philipines OR Phillipines OR Romania OR Rumania OR Roumania OR Russia OR Russian OR Rwanda OR Ruanda OR “Saint Lucia” OR “St Lucia” OR “Saint Vincent” OR “St Vincent” OR Grenadines OR Samoa OR Sao Tome OR Senegal OR Serbia OR Montenegro OR “Sierra Leone” OR “Sri Lanka” OR Ceylon OR “Solomon Islands” OR Somalia OR Sudan OR Suriname OR Surinam OR Swaziland OR Eswatini OR “South Africa” OR Syria OR Tajikistan OR Tadzhikistan OR Tadjikistan OR Tanzania OR Thailand OR Togo OR Tonga OR Tunisia OR Turkey OR Turkmenistan OR Tuvalu OR Uganda OR Ukraine OR Russia OR USSR OR “Soviet Union” OR Uzbekistan OR Uzbek OR Vanuatu OR Venezuela OR Vietnam OR “Viet Nam” OR “West Bank” OR Yemen OR Zambia OR Zimbabwe OR “Sub Saharan Africa” OR “SubSaharan Africa”)
**Combined search syntax**						
(hypertension OR hypertensive OR “high blood pressure” OR “blood pressure” OR diabetes OR diabet* OR dm2 OR niddm OR dm 2 OR t2d OR “dm type 2” OR “dm type II” OR dm1 OR iddm OR "dm 1" OR t1d OR “dm type 1” OR “dm type I” OR "Diabetes Mellitus") AND (“support group*” OR buddy OR “self-help group*” OR “peer group*” OR “informal group*” OR “social group” OR “Volunteers“ OR “Self-help groups" OR “Peer group“ OR “peer/microfinance“ OR “group medical visits“) AND (finance OR financing OR financial OR cash OR fund OR contribution OR support OR backing OR aid OR microfinance)						
**African Journals Online**						
Diabetes OR hypertension		Patient support groups		Financial component		Low and Middle Income Countries (LMICs)
(hypertension OR hypertensive OR “high blood pressure” OR “blood pressure” OR diabetes OR diabetic OR dm2 OR niddm OR "dm 2" OR t2d OR “dm type 2” OR “dm type II” OR dm1 OR iddm OR "dm 1" OR t1d OR “dm type 1” OR “dm type I” OR "Diabetes Mellitus")	AND	(“support group*” OR buddy OR “self-help group*” OR “peer group*” OR “informal group*” OR “social group” OR “Volunteers" OR “Self-help groups" OR “Peer group" OR OR "peer/microfinance" OR "group medical visits")	AND	(finance OR financing OR financial OR cash OR fund OR contribution OR support OR backing OR aid OR microfinance)	AND	(“emerging country” OR “emerging countries” OR “emerging nation” OR “emerging nations” OR “emerging population” OR “emerging populations” OR “developing country” OR “developing countries” OR “developing nation” OR “developing nations” OR “developing population” OR “developing populations” OR “developing world” OR “less developed country” OR “less developed countries” OR “less developed nation” OR “less developed nations” OR “less developed world” OR “lesser developed countries” OR “lesser developed nations” OR “under developed country” OR “under developed countries” OR “under developed nations” OR “under developed world” OR “underdeveloped country” OR “underdeveloped countries” OR “underdeveloped nation” OR “underdeveloped nations” OR “underdeveloped population” OR “underdeveloped populations” OR “underdeveloped world” OR “middle income country” OR “middle income countries” OR “middle income nation” OR “middle income nations” OR “middle income population” OR “middle income populations” OR “low income country” OR “low income countries” OR “low income nation” OR “low income nations” OR “low income population” OR “low income populations” OR “lower income country” OR “lower income countries” OR “lower income nations” OR “lower income population” OR “lower income populations” OR “underserved countries” OR “underserved nations” OR “underserved population” OR “underserved populations” OR “under served population” OR “under served populations” OR “deprived countries” OR “deprived population” OR “deprived populations” OR “poor country” OR “poor countries” OR “poor nation” OR “poor nations” OR “poor population” OR “poor populations” OR “poor world” OR “poorer countries” OR “poorer nations” OR “poorer population” OR “poorer populations” OR “developing economy” OR “developing economies” OR “less developed economy” OR “less developed economies” OR “underdeveloped economies” OR “middle income economy” OR “middle income economies” OR “low income economy” OR “low income economies” OR “lower income economies” OR “low gdp” OR “low gnp” OR “low gross domestic” OR “low gross national” OR “lower gdp” OR “lower gross domestic” OR lmic OR lmics OR “third world” OR “lami country” OR “lami countries” OR “transitional country” OR “transitional countries” OR Africa OR Asia OR Caribbean OR “West Indies” OR “South America” OR “Latin America” OR “Central America” OR “Atlantic Islands” OR “Pacific Islands” OR “Indian Ocean Islands” OR Afghanistan OR Albania OR Algeria OR Angola OR Argentina OR Armenia OR Azerbaijan OR Bangladesh OR Barbados OR Benin OR Byelarus OR Byelorussian OR Belarus OR Belorussian OR Belorussia OR Belize OR Bhutan OR Bolivia OR Bosnia OR Herzegovina OR Hercegovina OR Botswana OR Brasil OR Brazil

						OR Bulgaria OR “Burkina Faso” OR “Burkina Fasso” OR “Upper Volta” OR Burundi OR Urundi OR Cambodia OR “Khmer Republic” OR Kampuchea OR Cameroon OR Cameroons OR Cameron OR Cape Verde OR “Central African Republic” OR Chad OR China OR Colombia OR Comoros OR Comoro Islands OR Comores OR Mayotte OR Congo OR Zaire OR Costa Rica OR “Cote d'Ivoire” OR “Ivory Coast” OR Cuba OR Djibouti OR “French Somaliland” OR Dominica OR “Dominican Republic” OR “East Timor” OR “East Timur” OR “Timor Leste” OR Ecuador OR Egypt OR “El Salvador” OR Eritrea OR Ethiopia OR Fiji OR Gabon OR “Gabonese Republic” OR Gambia OR Gaza OR Georgia OR Ghana OR Gold Coast OR Grenada OR Guatemala OR Guinea OR Guam OR Guiana OR Guyana OR Haiti OR Honduras OR India OR Indonesia OR Iran OR Iraq OR Jamaica OR Jordan OR Kazakhstan OR Kenya OR Kiribati OR Kosovo OR Kyrgyzstan OR Kirghizia OR “Kyrgyz Republic” OR Kirghiz OR Kirgizstan OR “Lao PDR” OR Laos OR Latvia OR Lebanon OR Lesotho OR Liberia OR Libya OR Macedonia OR Madagascar OR Malaysia OR Malaya OR Malay OR Maldives OR Malawi OR Mali OR Marshall Islands OR Mauritania OR Mauritius OR Mexico OR Micronesia OR Moldova OR Moldovia OR Mongolia OR Montenegro OR Morocco OR Mozambique OR Myanmar OR Myanma OR Burma OR Namibia OR Nepal OR Nicaragua OR Niger OR Nigeria OR Pakistan OR Panama OR Paraguay OR Peru OR Philippines OR Philipines OR Phillipines OR Romania OR Rumania OR Roumania OR Russia OR Russian OR Rwanda OR Ruanda OR “Saint Lucia” OR “St Lucia” OR “Saint Vincent” OR “St Vincent” OR Grenadines OR Samoa OR Sao Tome OR Senegal OR Serbia OR Montenegro OR “Sierra Leone” OR “Sri Lanka” OR Ceylon OR “Solomon Islands” OR Somalia OR Sudan OR Suriname OR Surinam OR Swaziland OR Eswatini OR “South Africa” OR Syria OR Tajikistan OR Tadzhikistan OR Tadjikistan OR Tanzania OR Thailand OR Togo OR Tonga OR Tunisia OR Turkey OR Turkmenistan OR Tuvalu OR Uganda OR Ukraine OR Russia OR USSR OR “Soviet Union” OR Uzbekistan OR Uzbek OR Vanuatu OR Venezuela OR Vietnam OR “Viet Nam” OR “West Bank” OR Yemen OR Zambia OR Zimbabwe OR “Sub Saharan Africa” OR “SubSaharan Africa”)
**Combined search syntax**						
(hypertension OR hypertensive OR “high blood pressure” OR “blood pressure” OR diabetes OR diabetic OR dm2 OR niddm OR "dm 2" OR t2d OR “dm type 2” OR “dm type II” OR dm1 OR iddm OR "dm 1" OR t1d OR “dm type 1” OR “dm type I” OR "Diabetes Mellitus") AND (“support group*” OR buddy OR “self-help group*” OR “peer group*” OR “informal group*” OR “social group” OR “Volunteers" OR “Self-help groups" OR “Peer group" OR "peer/microfinance" OR "group medical visits") AND (finance OR financing OR financial OR cash OR fund OR contribution OR support OR backing OR aid OR microfinance)						
**CINAHL**						
Diabetes OR hypertension		Patient support groups		Financial component		Low and Middle Income Countries (LMICs)
(MH "hypertension" OR TI hypertension OR TI hypertensive OR TI “high blood pressure” OR TI “blood pressure” OR TI diabetes OR TI diabetic* OR TI dm2 OR TI niddm OR TI dm 2 OR TI t2d OR TI “dm type 2” OR TI “dm type II” OR TI dm1 OR TI iddm OR TI "dm 1" OR TI t1d OR TI “dm type 1” OR TI “dm type I” OR MH "Diabetes Mellitus" OR AB	AND	(TI “support group*” OR TI buddy OR TI “self-help group*” OR TI “peer group*” OR TI “informal group*” OR TI “social group” OR MH “Volunteers" OR MH “Self-help groups" OR MH “Peer group" OR AB “support group*” OR AB buddy OR AB “self-help group*” OR AB “peer group*” OR AB “informal group*” OR AB “social group” OR MH “Self-help groups" OR OR TI	AND	(TI financ* OR TI cash OR TI fund OR TI contribution OR TI support OR TI backing OR TI aid OR TI microfinance OR AB financ* OR AB cash OR AB fund OR AB contribution OR AB support OR AB backing OR AB aid OR AB microfinance)	AND	(“emerging country” OR “emerging countries” OR “emerging nation” OR “emerging nations” OR “emerging population” OR “emerging populations” OR TI "developing country" OR TI "developing countries" OR TI "developing nation" OR TI "developing nations" OR TI "developing population" OR TI "developing populations" OR TI "developing world" OR TI "less developed country" OR TI "less developed countries" OR TI "less developed nation" OR TI "less developed nations" OR TI "less developed world" OR TI "lesser developed countries" OR TI "lesser developed nations" OR TI "under developed country" OR TI "under developed countries" OR TI "under developed nations" OR TI "under developed world" OR TI "underdeveloped country" OR TI "underdeveloped countries" OR TI "underdeveloped nation" OR TI "underdeveloped nations" OR TI "underdeveloped population" OR TI "underdeveloped populations" OR TI "underdeveloped world" OR TI "middle income country" OR TI "middle income countries" OR TI "middle income nation" OR TI "middle income nations" OR TI "middle income population" OR TI "middle income populations" OR TI "low income country" OR TI "low income countries" OR TI "low income nation" OR TI "low income nations" OR TI "low income population" OR TI "low income populations" OR TI "lower income country" OR TI "lower income countries" OR TI "lower income nations" OR TI "lower income population" OR TI "lower income populations" OR TI "underserved countries" OR TI "underserved nations" OR TI "underserved population" OR TI "underserved populations" OR TI "under served population" OR TI "under

hypertension OR AB hypertensive OR AB “high blood pressure” OR AB “blood pressure” OR AB diabetes OR AB diabeABc* OR AB dm2 OR AB niddm OR AB dm 2 OR AB t2d OR AB “dm type 2” OR AB “dm type II” OR AB dm1 OR AB iddm OR AB dm 1 OR AB t1d OR AB “dm type 1” OR AB “dm type I”)		"peer/microfinance" OR TI "group medical visits" OR AB "peer/microfinance" OR AB "group medical visits")				served populations" OR TI "deprived countries" OR TI "deprived population" OR TI "deprived populations" OR TI "poor country" OR TI "poor countries" OR TI "poor nation" OR TI "poor nations" OR TI "poor population" OR TI "poor populations" OR TI "poor world" OR TI "poorer countries" OR TI "poorer population" OR TI "poorer populations" OR TI "developing economy" OR TI "developing economies" OR TI "less developed economy" OR TI "less developed economies" OR TI "underdeveloped economies" OR TI "middle income economy" OR TI "middle income economies" OR TI "low income economy" OR TI "low income economies" OR TI "lower income economies" OR TI "low gdp" OR TI "low gnp" OR TI "low gross domestic" OR TI "low gross national" OR TI "lower gdp" OR TI "lower gross domestic" OR TI lmic OR TI lmics OR TI "third world" OR TI "lami country" OR TI "lami countries" OR TI "transitional country" OR TI "transitional countries" OR TI Africa OR TI Asia OR TI Caribbean OR TI West Indies OR TI South America OR TI Latin America OR TI Central America OR TI "Atlantic Islands" OR TI "Pacific Islands" OR TI "Indian Ocean Islands" OR TI Afghanistan OR TI Albania OR TI Algeria OR TI Angola OR TI Argentina OR TI Armenia OR TI Azerbaijan OR TI Bangladesh OR TI Barbados OR TI Benin OR TI Byelarus OR TI Byelorussian OR TI Belarus OR TI Belorussian OR TI Belorussia OR TI Belize OR TI Bhutan OR TI Bolivia OR TI Bosnia OR TI Herzegovina OR TI Hercegovina OR TI Botswana OR TI Brasil OR TI Brazil OR TI Bulgaria OR TI Burkina Faso OR TI Burkina Fasso OR TI Upper Volta OR TI Burundi OR TI Urundi OR TI Cambodia OR TI Khmer Republic OR TI Kampuchea OR TI Cameroon OR TI Cameroons OR TI Cameron OR TI Cape Verde OR TI Central African Republic OR TI Chad OR TI China OR TI Colombia OR TI Comoros OR TI Comoro Islands OR TI Comores OR TI Mayotte OR TI Congo OR TI Zaire OR TI Costa Rica OR TI Cote d'Ivoire OR TI Ivory Coast OR TI Cuba OR TI Djibouti OR TI French Somaliland OR TI Dominica OR TI Dominican Republic OR TI East Timor OR TI East Timur OR TI TimOR TI Leste OR TI Ecuador OR TI Egypt OR TI El Salvador OR TI Eritrea OR TI Ethiopia OR TI Fiji OR TI Gabon OR TI Gabonese Republic OR TI Gambia OR TI Gaza OR TI Georgia OR TI Ghana OR TI Gold Coast OR TI Grenada OR TI Guatemala OR TI Guinea OR TI Guam OR TI Guiana OR TI Guyana OR TI Haiti OR TI Honduras OR TI India OR TI Indonesia OR TI Iran OR TI Iraq OR TI Jamaica OR TI Jordan OR TI Kazakhstan OR TI Kenya OR TI Kiribati OR TI Kosovo OR TI Kyrgyzstan OR TI Kirghizia OR TI Kyrgyz Republic OR TI Kirghiz OR TI Kirgizstan OR TI "Lao PDR" OR TI Laos OR TI Latvia OR TI Lebanon OR TI Lesotho OR TI Liberia OR TI Libya OR TI Macedonia OR TI Madagascar OR TI Malaysia OR TI Malaya OR TI Malay OR TI Maldives OR TI Malawi OR TI Mali OR TI Marshall Islands OR TI Mauritania OR TI Mauritius OR TI Mexico OR TI Micronesia OR TI Moldova OR TI Moldovia OR TI Mongolia OR TI Montenegro OR TI Morocco OR TI Mozambique OR TI Myanmar OR TI Myanma OR TI Burma OR TI Namibia OR TI Nepal OR TI Nicaragua OR TI Niger OR TI Nigeria OR TI Pakistan OR TI Panama OR TI Paraguay OR TI Peru OR TI Philippines OR TI Philipines OR TI Phillipines OR TI Romania OR TI Rumania OR TI Roumania OR TI Russia OR TI Russian OR TI Rwanda OR TI Ruanda OR TI Saint Lucia OR TI St Lucia OR TI Saint Vincent OR TI St Vincent OR TI Grenadines OR TI Samoa OR TI Sao Tome OR TI Senegal OR TI Serbia OR TI Montenegro OR TI Sierra Leone OR TI Sri Lanka OR TI Ceylon OR TI Solomon Islands OR TI Somalia OR TI Sudan OR TI Suriname OR TI Surinam OR TI Swaziland OR TI Eswatini OR TI South Africa OR TI Syria OR TI Tajikistan OR TI Tadzhikistan OR TI Tadjikistan OR TI Tanzania OR TI Thailand OR TI Togo OR TI Tonga OR TI Tunisia OR TI Turkey OR TI Turkmenistan OR TI Tuvalu OR TI Uganda OR TI Ukraine OR TI Russia OR TI USSR OR TI Soviet Union OR TI Uzbekistan OR TI Uzbek OR TI Vanuatu OR TI Venezuela OR TI Vietnam OR TI Viet Nam OR TI West Bank OR TI Yemen OR TI Zambia OR TI Zimbabwe OR AB "developing country" OR AB "developing countries" OR AB "developing nation" OR AB "developing nations" OR AB "developing population" OR AB "developing populations" OR AB "developing world" OR AB "less developed country" OR AB "less developed countries" OR AB "less developed nation" OR AB "less developed nations" OR AB "less developed world" OR AB "lesser developed countries" OR AB "lesser developed nations" OR AB "under developed country" OR AB "under developed countries" OR AB "under developed nations" OR AB "under developed world" OR AB "underdeveloped country" OR AB "underdeveloped countries" OR AB "underdeveloped nation" OR AB "underdeveloped nations" OR AB "underdeveloped population" OR AB

						"underdeveloped populations" OR AB "underdeveloped world" OR AB "middle income country" OR AB "middle income countries" OR AB "middle income nation" OR AB "middle income nations" OR AB "middle income population" OR AB "middle income populations" OR AB "low income country" OR AB "low income countries" OR AB "low income nation" OR AB "low income nations" OR AB "low income population" OR AB "low income populations" OR AB "lower income country" OR AB "lower income countries" OR AB "lower income nations" OR AB "lower income population" OR AB "lower income populations" OR AB "underserved countries" OR AB "underserved nations" OR AB "underserved population" OR AB "underserved populations" OR AB "under served population" OR AB "under served populations" OR AB "deprived countries" OR AB "deprived population" OR AB "deprived populations" OR AB "poor country" OR AB "poor countries" OR AB "poor nation" OR AB "poor nations" OR AB "poor population" OR AB "poor populations" OR AB "poor world" OR AB "poorer countries" OR AB "poorer population" OR AB "poorer populations" OR AB "developing economy" OR AB "developing economies" OR AB "less developed economy" OR AB "less developed economies" OR AB "underdeveloped economies" OR AB "middle income economy" OR AB "middle income economies" OR AB "low income economy" OR AB "low income economies" OR AB "lower income economies" OR AB "low gdp" OR AB "low gnp" OR AB "low gross domestic" OR AB "low gross national" OR AB "lower gdp" OR AB "lower gross domestic" OR AB lmic OR AB lmics OR AB "third world" OR AB "lami country" OR AB "lami countries" OR AB "transitional country" OR AB "transitional countries" OR AB Africa OR AB Asia OR AB Caribbean OR AB West Indies OR AB South America OR AB Latin America OR AB Central America OR AB "Atlantic Islands" OR AB "Pacific Islands" OR AB "Indian Ocean Islands" OR AB Afghanistan OR AB Albania OR AB Algeria OR AB Angola OR AB Argentina OR AB Armenia OR AB Azerbaijan OR AB Bangladesh OR AB Barbados OR AB Benin OR AB Byelarus OR AB Byelorussian OR AB Belarus OR AB Belorussian OR AB Belorussia OR AB Belize OR AB Bhutan OR AB Bolivia OR AB Bosnia OR AB Herzegovina OR AB Hercegovina OR AB Botswana OR AB Brasil OR AB Brazil OR AB Bulgaria OR AB Burkina Faso OR AB Burkina Fasso OR AB Upper Volta OR AB Burundi OR AB Urundi OR AB Cambodia OR AB Khmer Republic OR AB Kampuchea OR AB Cameroon OR AB Cameroons OR AB Cameron OR AB Cape Verde OR AB Central African Republic OR AB Chad OR AB China OR AB Colombia OR AB Comoros OR AB Comoro Islands OR AB Comores OR AB Mayotte OR AB Congo OR AB Zaire OR AB Costa Rica OR AB Cote d'Ivoire OR AB Ivory Coast OR AB Cuba OR AB Djibouti OR AB French Somaliland OR AB Dominica OR AB Dominican Republic OR AB East Timor OR AB East Timur OR AB TimOR AB Leste OR AB Ecuador OR AB Egypt OR AB El Salvador OR AB Eritrea OR AB Ethiopia OR AB Fiji OR AB Gabon OR AB Gabonese Republic OR AB Gambia OR AB Gaza OR AB Georgia OR AB Ghana OR AB Gold Coast OR AB Grenada OR AB Guatemala OR AB Guinea OR AB Guam OR AB Guiana OR AB Guyana OR AB Haiti OR AB Honduras OR AB India OR AB Indonesia OR AB Iran OR AB Iraq OR AB Jamaica OR AB Jordan OR AB Kazakhstan OR AB Kenya OR AB Kiribati OR AB Kosovo OR AB Kyrgyzstan OR AB Kirghizia OR AB Kyrgyz Republic OR AB Kirghiz OR AB Kirgizstan OR AB "Lao PDR" OR AB Laos OR AB Latvia OR AB Lebanon OR AB Lesotho OR AB Liberia OR AB Libya OR AB Macedonia OR AB Madagascar OR AB Malaysia OR AB Malaya OR AB Malay OR AB Maldives OR AB Malawi OR AB Mali OR AB Marshall Islands OR AB Mauritania OR AB Mauritius OR AB Mexico OR AB Micronesia OR AB Moldova OR AB Moldovia OR AB Mongolia OR AB Montenegro OR AB Morocco OR AB Mozambique OR AB Myanmar OR AB Myanma OR AB Burma OR AB Namibia OR AB Nepal OR AB Nicaragua OR AB Niger OR AB Nigeria OR AB Pakistan OR AB Panama OR AB Paraguay OR AB Peru OR AB Philippines OR AB Philipines OR AB Phillipines OR AB Romania OR AB Rumania OR AB Roumania OR AB Russia OR AB Russian OR AB Rwanda OR AB Ruanda OR AB Saint Lucia OR AB St Lucia OR AB Saint Vincent OR AB St Vincent OR AB Grenadines OR AB Samoa OR AB Sao Tome OR AB Senegal OR AB Serbia OR AB Montenegro OR AB Sierra Leone OR AB Sri Lanka OR AB Ceylon OR AB Solomon Islands OR AB Somalia OR AB Sudan OR AB Suriname OR AB Surinam OR AB Swaziland OR AB Eswatini OR AB South Africa OR AB Syria OR AB Tajikistan OR AB Tadzhikistan OR AB Tadjikistan OR AB Tanzania OR AB Thailand OR AB Togo OR AB Tonga OR AB Tunisia OR AB Turkey OR AB Turkmenistan OR AB

						Tuvalu OR AB Uganda OR AB Ukraine OR AB Russia OR AB USSR OR AB Soviet Union OR AB Uzbekistan OR AB Uzbek OR AB Vanuatu OR AB Venezuela OR AB Vietnam OR AB Viet Nam OR AB West Bank OR AB Yemen OR AB Zambia OR AB Zimbabwe OR MH Developing Countries OR MH Africa OR MH Africa, Northern OR MH Africa South of the Sahara OR MH Africa, Central OR MH Africa, Eastern OR MH Africa, Southern OR MH Africa, Western OR MH Asia OR MH Asia, Central OR MH Asia, Southeastern OR MH Asia, Western OR MH Caribbean Region OR MH West Indies OR MH South America OR MH Latin America OR MH Central America OR MH "Atlantic Islands" OR MH "Pacific Islands" OR MH "Indian Ocean Islands" OR MH Afghanistan OR MH Albania OR MH Algeria OR MH American Samoa OR MH Angola OR MH Argentina OR MH Armenia OR MH Azerbaijan OR MH Bangladesh OR MH Barbados OR MH Benin OR MH "Republic of Belarus" OR MH Belize OR MH Bhutan OR MH Bolivia OR MH Bosnia-Herzegovina OR MH Botswana OR MH Brazil OR MH Bulgaria OR MH Burkina Faso OR MH Burundi OR MH Cambodia OR MH Cameroon OR MH Cape Verde OR MH Central African Republic OR MH Chad OR MH China OR MH Colombia OR MH Comoros OR MH Congo OR MH Costa Rica OR MH Cote d'Ivoire OR MH Croatia OR MH Cuba OR MH Slovakia OR MH Djibouti OR MH "Democratic Republic of the Congo" OR MH Dominica OR MH Dominican Republic OR MH East Timor OR MH Ecuador OR MH Egypt OR MH El Salvador OR MH Eritrea OR MH Ethiopia OR MH "Equatorial Guinea" OR MH Fiji OR MH "French Guiana" OR MH Gabon OR MH Gambia OR MH Ghana OR MH Greece OR MH Grenada OR MH Guatemala OR MH Guinea OR MH Guinea-Bissau OR MH Guam OR MH Guyana OR MH Haiti OR MH Honduras OR MH "Independent State of Samoa" OR MH India OR MH Indonesia OR MH Iran OR MH Iraq OR MH Jamaica OR MH Jordan OR MH Kazakhstan OR MH Kenya OR MH Korea OR MH Kyrgyzstan OR MH Laos OR MH Latvia OR MH Lebanon OR MH Lesotho OR MH Liberia OR MH Libya OR MH "Macedonia (Republic)" OR MH Madagascar OR MH Malawi OR MH Malaysia OR MH Mali OR MH Malta OR MH Mauritania OR MH Mauritius OR MH "Melanesia" OR MH Mexico OR MH Micronesia OR MH Moldova OR MH Mongolia OR MH Montenegro OR MH Morocco OR MH Mozambique OR MH Myanmar OR MH Namibia OR MH Nepal OR MH Nicaragua OR MH Niger OR MH Nigeria OR MH Pakistan OR MH Panama OR MH Papua New Guinea OR MH Paraguay OR MH Peru OR MH Philippines OR MH "Republic of Korea" OR MH Romania OR MH Russia OR MH "Russia (Pre-1917)" OR MH Rwanda OR MH Saint Lucia OR MH "Saint Vincent and the Grenadines" OR MH Samoa OR MH Senegal OR MH Serbia OR MH Montenegro OR MH Sierra Leone OR MH Slovenia OR MH Sri Lanka OR MH Somalia OR MH South Africa OR MH Sudan OR MH Suriname OR MH Swaziland OR MH Syria OR MH Tajikistan OR MH Tanzania OR MH Thailand OR MH Togo OR MH Tonga OR MH Tunisia OR MH Turkey OR MH Turkmenistan OR MH Uganda OR MH Ukraine OR MH Uruguay OR MH USSR OR MH Uzbekistan OR MH Vanuatu OR MH Venezuela OR MH Vietnam OR MH Yemen OR MH Yugoslavia OR MH Zambia OR MH Zimbabwe OR “Sub Saharan Africa " OR “SubSaharan Africa ")
**Combined search syntax**						
(MH "hypertension" OR TI hypertension OR TI hypertensive OR TI “high blood pressure” OR TI “blood pressure” OR TI diabetes OR TI diabetic* OR TI dm2 OR TI niddm OR TI dm 2 OR TI t2d OR TI “dm type 2” OR TI “dm type II” OR TI dm1 OR TI iddm OR TI "dm 1" OR TI t1d OR TI “dm type 1” OR TI “dm type I” OR MH "Diabetes Mellitus" OR AB hypertension OR AB hypertensive OR AB “high blood pressure” OR AB “blood pressure” OR AB diabetes OR AB diabeABc* OR AB dm2 OR AB niddm OR AB dm 2 OR AB t2d OR AB “dm type 2” OR AB “dm type II” OR AB dm1 OR AB iddm OR AB dm 1 OR AB t1d OR AB “dm type 1” OR AB “dm type I”) AND (TI “support group*” OR TI buddy OR TI “self-help group*” OR TI “peer group*” OR TI “informal group*” OR TI “social group” OR MH “Volunteers" OR MH “Self-help groups" OR MH “Peer group" OR AB “support group*” OR AB buddy OR AB “self-help group*” OR AB “peer group*” OR AB “informal group*” OR AB “social group” OR MH “Self-help groups" OR OR TI "peer/microfinance" OR TI "group medical visits" OR AB "peer/microfinance" OR AB "group medical visits") AND (TI financ* OR TI cash OR TI fund OR TI contribution OR TI support OR TI backing OR TI aid OR TI microfinance OR AB financ* OR AB cash OR AB fund OR AB contribution OR AB support OR AB backing OR AB aid OR AB microfinance) AND (“emerging country” OR “emerging countries” OR “emerging nation” OR “emerging nations” OR “emerging population” OR “emerging populations” OR TI "developing country" OR TI "developing countries" OR TI "developing nation" OR TI "developing nations" OR TI "developing population" OR TI "developing populations" OR TI "developing world" OR TI "less developed country" OR TI "less developed countries" OR TI "less developed nation" OR TI "less developed nations" OR TI "less developed world" OR TI "lesser developed countries" OR TI "lesser developed nations" OR TI "under developed country" OR TI "under developed countries" OR TI "under developed nations" OR TI "under developed world" OR TI "underdeveloped country" OR TI "underdeveloped countries" OR TI "underdeveloped nation" OR TI "underdeveloped nations" OR TI "underdeveloped population" OR TI "underdeveloped populations" OR TI "underdeveloped world" OR TI "middle income country" OR TI "middle income countries" OR TI "middle income nation" OR TI "middle income nations" OR TI "middle income population" OR TI "middle income						

populations" OR TI "low income country" OR TI "low income countries" OR TI "low income nation" OR TI "low income nations" OR TI "low income population" OR TI "low income populations" OR TI "lower income country" OR TI "lower income countries" OR TI "lower income nations" OR TI "lower income population" OR TI "lower income populations" OR TI "underserved countries" OR TI "underserved nations" OR TI "underserved population" OR TI "underserved populations" OR TI "under served population" OR TI "under served populations" OR TI "deprived countries" OR TI "deprived population" OR TI "deprived populations" OR TI "poor country" OR TI "poor countries" OR TI "poor nation" OR TI "poor nations" OR TI "poor population" OR TI "poor populations" OR TI "poor world" OR TI "poorer countries" OR TI "poorer population" OR TI "poorer populations" OR TI "developing economy" OR TI "developing economies" OR TI "less developed economy" OR TI "less developed economies" OR TI "underdeveloped economies" OR TI "middle income economy" OR TI "middle income economies" OR TI "low income economy" OR TI "low income economies" OR TI "lower income economies" OR TI "low gdp" OR TI "low gnp" OR TI "low gross domestic" OR TI "low gross national" OR TI "lower gdp" OR TI "lower gross domestic" OR TI lmic OR TI lmics OR TI "third world" OR TI "lami country" OR TI "lami countries" OR TI "transitional country" OR TI "transitional countries" OR TI Africa OR TI Asia OR TI Caribbean OR TI West Indies OR TI South America OR TI Latin America OR TI Central America OR TI "Atlantic Islands" OR TI "Pacific Islands" OR TI "Indian Ocean Islands" OR TI Afghanistan OR TI Albania OR TI Algeria OR TI Angola OR TI Argentina OR TI Armenia OR TI Azerbaijan OR TI Bangladesh OR TI Barbados OR TI Benin OR TI Byelarus OR TI Byelorussian OR TI Belarus OR TI Belorussian OR TI Belorussia OR TI Belize OR TI Bhutan OR TI Bolivia OR TI Bosnia OR TI Herzegovina OR TI Hercegovina OR TI Botswana OR TI Brasil OR TI Brazil OR TI Bulgaria OR TI Burkina Faso OR TI Burkina Fasso OR TI Upper Volta OR TI Burundi OR TI Urundi OR TI Cambodia OR TI Khmer Republic OR TI Kampuchea OR TI Cameroon OR TI Cameroons OR TI Cameron OR TI Cape Verde OR TI Central African Republic OR TI Chad OR TI China OR TI Colombia OR TI Comoros OR TI Comoro Islands OR TI Comores OR TI Mayotte OR TI Congo OR TI Zaire OR TI Costa Rica OR TI Cote d'Ivoire OR TI Ivory Coast OR TI Cuba OR TI Djibouti OR TI French Somaliland OR TI Dominica OR TI Dominican Republic OR TI East Timor OR TI East Timur OR TI TimOR TI Leste OR TI Ecuador OR TI Egypt OR TI El Salvador OR TI Eritrea OR TI Ethiopia OR TI Fiji OR TI Gabon OR TI Gabonese Republic OR TI Gambia OR TI Gaza OR TI Georgia OR TI Ghana OR TI Gold Coast OR TI Grenada OR TI Guatemala OR TI Guinea OR TI Guam OR TI Guiana OR TI Guyana OR TI Haiti OR TI Honduras OR TI India OR TI Indonesia OR TI Iran OR TI Iraq OR TI Jamaica OR TI Jordan OR TI Kazakhstan OR TI Kenya OR TI Kiribati OR TI Kosovo OR TI Kyrgyzstan OR TI Kirghizia OR TI Kyrgyz Republic OR TI Kirghiz OR TI Kirgizstan OR TI "Lao PDR" OR TI Laos OR TI Latvia OR TI Lebanon OR TI Lesotho OR TI Liberia OR TI Libya OR TI Macedonia OR TI Madagascar OR TI Malaysia OR TI Malaya OR TI Malay OR TI Maldives OR TI Malawi OR TI Mali OR TI Marshall Islands OR TI Mauritania OR TI Mauritius OR TI Mexico OR TI Micronesia OR TI Moldova OR TI Moldovia OR TI Mongolia OR TI Montenegro OR TI Morocco OR TI Mozambique OR TI Myanmar OR TI Myanma OR TI Burma OR TI Namibia OR TI Nepal OR TI Nicaragua OR TI Niger OR TI Nigeria OR TI Pakistan OR TI Panama OR TI Paraguay OR TI Peru OR TI Philippines OR TI Philipines OR TI Phillipines OR TI Romania OR TI Rumania OR TI Roumania OR TI Russia OR TI Russian OR TI Rwanda OR TI Ruanda OR TI Saint Lucia OR TI St Lucia OR TI Saint Vincent OR TI St Vincent OR TI Grenadines OR TI Samoa OR TI Sao Tome OR TI Senegal OR TI Serbia OR TI Montenegro OR TI Sierra Leone OR TI Sri Lanka OR TI Ceylon OR TI Solomon Islands OR TI Somalia OR TI Sudan OR TI Suriname OR TI Surinam OR TI Swaziland OR TI Eswatini OR TI South Africa OR TI Syria OR TI Tajikistan OR TI Tadzhikistan OR TI Tadjikistan OR TI Tanzania OR TI Thailand OR TI Togo OR TI Tonga OR TI Tunisia OR TI Turkey OR TI Turkmenistan OR TI Tuvalu OR TI Uganda OR TI Ukraine OR TI Russia OR TI USSR OR TI Soviet Union OR TI Uzbekistan OR TI Uzbek OR TI Vanuatu OR TI Venezuela OR TI Vietnam OR TI Viet Nam OR TI West Bank OR TI Yemen OR TI Zambia OR TI Zimbabwe OR AB "developing country" OR AB "developing countries" OR AB "developing nation" OR AB "developing nations" OR AB "developing population" OR AB "developing populations" OR AB "developing world" OR AB "less developed country" OR AB "less developed countries" OR AB "less developed nation" OR AB "less developed nations" OR AB "less developed world" OR AB "lesser developed countries" OR AB "lesser developed nations" OR AB "under developed country" OR AB "under developed countries" OR AB "under developed nations" OR AB "under developed world" OR AB "underdeveloped country" OR AB "underdeveloped countries" OR AB "underdeveloped nation" OR AB "underdeveloped nations" OR AB "underdeveloped population" OR AB "underdeveloped populations" OR AB "underdeveloped world" OR AB "middle income country" OR AB "middle income countries" OR AB "middle income nation" OR AB "middle income nations" OR AB "middle income population" OR AB "middle income populations" OR AB "low income country" OR AB "low income countries" OR AB "low income nation" OR AB "low income nations" OR AB "low income population" OR AB "low income populations" OR AB "lower income country" OR AB "lower income countries" OR AB "lower income nations" OR AB "lower income population" OR AB "lower income populations" OR AB "underserved countries" OR AB "underserved nations" OR AB "underserved population" OR AB "underserved populations" OR AB "under served population" OR AB "under served populations" OR AB "deprived countries" OR AB "deprived population" OR AB "deprived populations" OR AB "poor country" OR AB "poor countries" OR AB "poor nation" OR AB "poor nations" OR AB "poor population" OR AB "poor populations" OR AB "poor world" OR AB "poorer countries" OR AB "poorer population" OR AB "poorer populations" OR AB "developing economy" OR AB "developing economies" OR AB "less developed economy" OR AB "less developed economies" OR AB "underdeveloped economies" OR AB "middle income economy" OR AB "middle income economies" OR AB "low income economy" OR AB "low income economies" OR AB "lower income economies" OR AB "low gdp" OR AB "low gnp" OR AB "low gross domestic" OR AB "low gross national" OR AB "lower gdp" OR AB "lower gross domestic" OR AB lmic OR AB lmics OR AB "third world" OR AB "lami country" OR AB "lami countries" OR AB "transitional country" OR AB "transitional countries" OR AB Africa OR AB Asia OR AB Caribbean OR AB West Indies OR AB South America OR AB Latin America OR AB Central America OR AB "Atlantic Islands" OR AB "Pacific Islands" OR AB "Indian Ocean Islands" OR AB Afghanistan OR AB Albania OR AB Algeria OR AB Angola OR AB Argentina OR AB Armenia OR AB Azerbaijan OR AB Bangladesh OR AB Barbados OR AB Benin OR AB Byelarus OR AB Byelorussian OR AB Belarus OR AB Belorussian OR AB Belorussia OR AB Belize OR AB Bhutan OR AB Bolivia OR AB Bosnia OR AB Herzegovina OR AB Hercegovina OR AB Botswana OR AB Brasil OR AB Brazil OR AB Bulgaria OR AB Burkina Faso OR AB Burkina Fasso OR AB Upper Volta OR AB Burundi OR AB Urundi OR AB Cambodia OR AB Khmer Republic OR AB Kampuchea OR AB Cameroon OR AB Cameroons OR AB Cameron OR AB Cape Verde OR AB Central African Republic OR AB Chad OR AB China OR AB Colombia OR AB Comoros OR AB Comoro Islands OR AB Comores OR AB Mayotte OR AB Congo OR AB Zaire OR AB Costa Rica OR AB Cote d'Ivoire OR AB Ivory Coast OR AB Cuba OR AB Djibouti OR AB French Somaliland OR AB Dominica OR AB Dominican Republic OR AB East Timor OR AB East Timur OR AB TimOR AB Leste OR AB Ecuador OR AB Egypt OR AB El Salvador OR AB Eritrea OR AB Ethiopia OR AB Fiji OR AB Gabon OR AB Gabonese Republic OR AB Gambia OR AB Gaza OR AB Georgia OR AB Ghana OR AB Gold Coast OR AB Grenada OR AB Guatemala OR AB Guinea OR AB Guam OR AB Guiana OR AB Guyana OR AB Haiti OR AB Honduras OR AB India OR AB Indonesia OR AB Iran OR AB Iraq OR AB Jamaica OR AB Jordan OR AB Kazakhstan OR AB Kenya OR AB Kiribati OR AB Kosovo OR AB Kyrgyzstan OR AB Kirghizia OR AB Kyrgyz Republic OR AB Kirghiz OR AB Kirgizstan OR AB "Lao PDR" OR AB Laos OR AB Latvia OR AB Lebanon OR AB Lesotho OR AB Liberia OR AB Libya OR AB Macedonia OR AB Madagascar OR AB Malaysia OR AB Malaya OR AB Malay OR AB Maldives OR AB Malawi OR AB Mali OR AB Marshall Islands OR AB Mauritania OR AB Mauritius OR AB Mexico OR AB Micronesia OR AB Moldova OR AB Moldovia OR AB Mongolia OR AB Montenegro OR AB Morocco OR AB Mozambique OR AB Myanmar OR AB Myanma OR AB Burma OR AB Namibia OR AB Nepal OR AB Nicaragua OR AB Niger OR AB Nigeria OR AB Pakistan OR AB Panama OR AB Paraguay OR AB Peru OR AB Philippines OR AB Philipines OR AB Phillipines

OR AB Romania OR AB Rumania OR AB Roumania OR AB Russia OR AB Russian OR AB Rwanda OR AB Ruanda OR AB Saint Lucia OR AB St Lucia OR AB Saint Vincent OR AB St Vincent OR AB Grenadines OR AB Samoa OR AB Sao Tome OR AB Senegal OR AB Serbia OR AB Montenegro OR AB Sierra Leone OR AB Sri Lanka OR AB Ceylon OR AB Solomon Islands OR AB Somalia OR AB Sudan OR AB Suriname OR AB Surinam OR AB Swaziland OR AB Eswatini OR AB South Africa OR AB Syria OR AB Tajikistan OR AB Tadzhikistan OR AB Tadjikistan OR AB Tanzania OR AB Thailand OR AB Togo OR AB Tonga OR AB Tunisia OR AB Turkey OR AB Turkmenistan OR AB Tuvalu OR AB Uganda OR AB Ukraine OR AB Russia OR AB USSR OR AB Soviet Union OR AB Uzbekistan OR AB Uzbek OR AB Vanuatu OR AB Venezuela OR AB Vietnam OR AB Viet Nam OR AB West Bank OR AB Yemen OR AB Zambia OR AB Zimbabwe OR MH Developing Countries OR MH Africa OR MH Africa, Northern OR MH Africa South of the Sahara OR MH Africa, Central OR MH Africa, Eastern OR MH Africa, Southern OR MH Africa, Western OR MH Asia OR MH Asia, Central OR MH Asia, Southeastern OR MH Asia, Western OR MH Caribbean Region OR MH West Indies OR MH South America OR MH Latin America OR MH Central America OR MH "Atlantic Islands" OR MH "Pacific Islands" OR MH "Indian Ocean Islands" OR MH Afghanistan OR MH Albania OR MH Algeria OR MH American Samoa OR MH Angola OR MH Argentina OR MH Armenia OR MH Azerbaijan OR MH Bangladesh OR MH Barbados OR MH Benin OR MH "Republic of Belarus" OR MH Belize OR MH Bhutan OR MH Bolivia OR MH Bosnia-Herzegovina OR MH Botswana OR MH Brazil OR MH Bulgaria OR MH Burkina Faso OR MH Burundi OR MH Cambodia OR MH Cameroon OR MH Cape Verde OR MH Central African Republic OR MH Chad OR MH China OR MH Colombia OR MH Comoros OR MH Congo OR MH Costa Rica OR MH Cote d'Ivoire OR MH Croatia OR MH Cuba OR MH Slovakia OR MH Djibouti OR MH "Democratic Republic of the Congo" OR MH Dominica OR MH Dominican Republic OR MH East Timor OR MH Ecuador OR MH Egypt OR MH El Salvador OR MH Eritrea OR MH Ethiopia OR MH "Equatorial Guinea" OR MH Fiji OR MH "French Guiana" OR MH Gabon OR MH Gambia OR MH Ghana OR MH Greece OR MH Grenada OR MH Guatemala OR MH Guinea OR MH Guinea-Bissau OR MH Guam OR MH Guyana OR MH Haiti OR MH Honduras OR MH "Independent State of Samoa" OR MH India OR MH Indonesia OR MH Iran OR MH Iraq OR MH Jamaica OR MH Jordan OR MH Kazakhstan OR MH Kenya OR MH Korea OR MH Kyrgyzstan OR MH Laos OR MH Latvia OR MH Lebanon OR MH Lesotho OR MH Liberia OR MH Libya OR MH "Macedonia (Republic)" OR MH Madagascar OR MH Malawi OR MH Malaysia OR MH Mali OR MH Malta OR MH Mauritania OR MH Mauritius OR MH "Melanesia" OR MH Mexico OR MH Micronesia OR MH Moldova OR MH Mongolia OR MH Montenegro OR MH Morocco OR MH Mozambique OR MH Myanmar OR MH Namibia OR MH Nepal OR MH Nicaragua OR MH Niger OR MH Nigeria OR MH Pakistan OR MH Panama OR MH Papua New Guinea OR MH Paraguay OR MH Peru OR MH Philippines OR MH "Republic of Korea" OR MH Romania OR MH Russia OR MH "Russia (Pre-1917)" OR MH Rwanda OR MH Saint Lucia OR MH "Saint Vincent and the Grenadines" OR MH Samoa OR MH Senegal OR MH Serbia OR MH Montenegro OR MH Sierra Leone OR MH Slovenia OR MH Sri Lanka OR MH Somalia OR MH South Africa OR MH Sudan OR MH Suriname OR MH Swaziland OR MH Syria OR MH Tajikistan OR MH Tanzania OR MH Thailand OR MH Togo OR MH Tonga OR MH Tunisia OR MH Turkey OR MH Turkmenistan OR MH Uganda OR MH Ukraine OR MH Uruguay OR MH USSR OR MH Uzbekistan OR MH Vanuatu OR MH Venezuela OR MH Vietnam OR MH Yemen OR MH Yugoslavia OR MH Zambia OR MH Zimbabwe OR “Sub Saharan Africa " OR “SubSaharan Africa ")						
**African Index Medicus**						
Diabetes OR hypertension		Patient support groups		Financial component		Low and Middle Income Countries (LMICs)
(hypertension OR hypertensive OR high blood pressure OR blood pressure OR diabetes OR diabetic OR dm2 OR niddm OR dm 2 OR t2d OR dm type 2 OR dm type II OR dm1 OR iddm OR dm 1 OR t1d OR dm type 1 OR dm type I OR Diabetes Mellitus)	AND	(support group* OR buddy OR self-help group* OR peer group* OR informal group* OR social group OR Volunteers OR Self-help groups OR Peer group OR peer/microfinance OR group medical visits)	AND	(finance OR financing OR financial OR cash OR fund OR contribution OR support OR backing OR aid OR microfinance)	AND	(emerging country OR emerging countries OR emerging nation OR emerging nations OR emerging population OR emerging populations OR developing country OR developing countries OR developing nation OR developing nations OR developing population OR developing populations OR developing world OR less developed country OR less developed countries OR less developed nation OR less developed nations OR less developed world OR lesser developed countries OR lesser developed nations OR under developed country OR under developed countries OR under developed nations OR under developed world OR underdeveloped country OR underdeveloped countries OR underdeveloped nation OR underdeveloped nations OR underdeveloped population OR underdeveloped populations OR underdeveloped world OR middle income country OR middle income countries OR middle income nation OR middle income nations OR middle income population OR middle income populations OR low income country OR low income countries OR low income nation OR low income nations OR low income population OR low income populations OR lower income country OR lower income countries OR lower income nations OR lower income population OR lower income populations OR underserved countries OR underserved nations OR underserved population OR underserved populations OR under served population OR under served populations OR deprived countries OR deprived population OR deprived populations OR poor country OR poor countries OR poor nation OR poor nations OR poor population OR poor populations OR poor world OR poorer countries OR poorer nations OR poorer population OR poorer populations OR developing economy OR developing economies OR less developed economy OR less developed economies OR underdeveloped economies OR middle income economy OR middle income economies OR low income economy OR low income economies OR lower income economies OR low gdp OR low gnp OR low gross domestic OR low gross national OR lower gdp OR lower gross domestic OR lmic OR lmics OR third world OR lami country OR lami countries OR transitional country OR transitional countries OR Africa OR Asia OR Caribbean OR West Indies OR South America OR Latin America OR Central

						America OR Atlantic Islands OR Pacific Islands OR Indian Ocean Islands OR Afghanistan OR Albania OR Algeria OR Angola OR Argentina OR Armenia OR Azerbaijan OR Bangladesh OR Barbados OR Benin OR Byelarus OR Byelorussian OR Belarus OR Belorussian OR Belorussia OR Belize OR Bhutan OR Bolivia OR Bosnia OR Herzegovina OR Hercegovina OR Botswana OR Brasil OR Brazil OR Bulgaria OR Burkina Faso OR Burkina Fasso OR Upper Volta OR Burundi OR Urundi OR Cambodia OR Khmer Republic OR Kampuchea OR Cameroon OR Cameroons OR Cameron OR Cape Verde OR Central African Republic OR Chad OR China OR Colombia OR Comoros OR Comoro Islands OR Comores OR Mayotte OR Congo OR Zaire OR Costa Rica OR Cote d'Ivoire OR Ivory Coast OR Cuba OR Djibouti OR French Somaliland OR Dominica OR Dominican Republic OR East Timor OR East Timur OR Timor Leste OR Ecuador OR Egypt OR El Salvador OR Eritrea OR Ethiopia OR Fiji OR Gabon OR Gabonese Republic OR Gambia OR Gaza OR Georgia OR Ghana OR Gold Coast OR Grenada OR Guatemala OR Guinea OR Guam OR Guiana OR Guyana OR Haiti OR Honduras OR India OR Indonesia OR Iran OR Iraq OR Jamaica OR Jordan OR Kazakhstan OR Kenya OR Kiribati OR Kosovo OR Kyrgyzstan OR Kirghizia OR Kyrgyz Republic OR Kirghiz OR Kirgizstan OR Lao PDR OR Laos OR Latvia OR Lebanon OR Lesotho OR Liberia OR Libya OR Macedonia OR Madagascar OR Malaysia OR Malaya OR Malay OR Maldives OR Malawi OR Mali OR Marshall Islands OR Mauritania OR Mauritius OR Mexico OR Micronesia OR Moldova OR Moldovia OR Mongolia OR Montenegro OR Morocco OR Mozambique OR Myanmar OR Myanma OR Burma OR Namibia OR Nepal OR Nicaragua OR Niger OR Nigeria OR Pakistan OR Panama OR Paraguay OR Peru OR Philippines OR Philipines OR Phillipines OR Romania OR Rumania OR Roumania OR Russia OR Russian OR Rwanda OR Ruanda OR Saint Lucia OR St Lucia OR Saint Vincent OR St Vincent OR Grenadines OR Samoa OR Sao Tome OR Senegal OR Serbia OR Montenegro OR Sierra Leone OR Sri Lanka OR Ceylon OR Solomon Islands OR Somalia OR Sudan OR Suriname OR Surinam OR Swaziland OR Eswatini OR South Africa OR Syria OR Tajikistan OR Tadzhikistan OR Tadjikistan OR Tanzania OR Thailand OR Togo OR Tonga OR Tunisia OR Turkey OR Turkmenistan OR Tuvalu OR Uganda OR Ukraine OR Russia OR USSR OR Soviet Union OR Uzbekistan OR Uzbek OR Vanuatu OR Venezuela OR Vietnam OR Viet Nam OR West Bank OR Yemen OR Zambia OR Zimbabwe OR Sub Saharan Africa OR SubSaharan Africa)
**Combined search syntax**						
(hypertension OR hypertensive OR high blood pressure OR blood pressure OR diabetes OR diabetic OR dm2 OR niddm OR dm 2 OR t2d OR dm type 2 OR dm type II OR dm1 OR iddm OR dm 1 OR t1d OR dm type 1 OR dm type I OR Diabetes Mellitus) AND (support group* OR buddy OR self-help group* OR peer group* OR informal group* OR social group OR Volunteers OR Self-help groups OR Peer group OR peer microfinance OR group medical visits) AND (finance OR financing OR financial OR cash OR fund OR contribution OR support OR backing OR aid OR microfinance)						
**Open Grey**						
Diabetes OR hypertension		Patient support groups		Financial component		Low and Middle Income Countries (LMICs)
(hypertens* OR “high blood pressure” OR “blood pressure” OR diabet* OR dm2 OR niddm OR dm 2 OR t2d OR “dm type 2” OR “dm type II” OR dm1 OR iddm OR “dm 1” OR t1d OR “dm type 1” OR “dm type I” OR "Diabetes Mellitus")	AND	(“support group*” OR buddy OR “self-help group*” OR “peer group*” OR “informal group*” OR “social group” OR volunteer* OR “Self-help groups" OR "peer/microfinance" OR "group medical visits")	AND	(financ* OR cash OR fund OR contribution OR support OR backing OR aid OR microfinance)	AND	(“emerging country” OR “emerging countries” OR “emerging nation” OR “emerging nations” OR “emerging population” OR “emerging populations” OR “developing country” OR “developing countries” OR “developing nation” OR “developing nations” OR “developing population” OR “developing populations” OR “developing world” OR “less developed country” OR “less developed countries” OR “less developed nation” OR “less developed nations” OR “less developed world” OR “lesser developed countries” OR “lesser developed nations” OR “under developed country” OR “under developed countries” OR “under developed nations” OR “under developed world” OR “underdeveloped country” OR “underdeveloped countries” OR “underdeveloped nation” OR “underdeveloped nations” OR “underdeveloped population” OR “underdeveloped populations” OR “underdeveloped world” OR “middle income country” OR “middle income countries” OR “middle income nation” OR “middle income nations” OR “middle income population” OR “middle income populations” OR “low income country” OR “low income countries” OR “low income nation” OR “low income nations” OR “low income population” OR “low income populations” OR “lower income country” OR “lower income countries” OR “lower income nations” OR “lower income population” OR

						“lower income populations” OR “underserved countries” OR “underserved nations” OR “underserved population” OR “underserved populations” OR “under served population” OR “under served populations” OR “deprived countries” OR “deprived population” OR “deprived populations” OR “poor country” OR “poor countries” OR “poor nation” OR “poor nations” OR “poor population” OR “poor populations” OR “poor world” OR “poorer countries” OR “poorer nations” OR “poorer population” OR “poorer populations” OR “developing economy” OR “developing economies” OR “less developed economy” OR “less developed economies” OR “underdeveloped economies” OR “middle income economy” OR “middle income economies” OR “low income economy” OR “low income economies” OR “lower income economies” OR “low gdp” OR “low gnp” OR “low gross domestic” OR “low gross national” OR “lower gdp” OR “lower gross domestic” OR lmic OR lmics OR “third world” OR “lami country” OR “lami countries” OR “transitional country” OR “transitional countries” OR Africa OR Asia OR Caribbean OR “West Indies” OR “South America” OR “Latin America” OR “Central America” OR “Atlantic Islands” OR “Pacific Islands” OR “Indian Ocean Islands” OR Afghanistan OR Albania OR Algeria OR Angola OR Argentina OR Armenia OR Azerbaijan OR Bangladesh OR Barbados OR Benin OR Byelarus OR Byelorussian OR Belarus OR Belorussian OR Belorussia OR Belize OR Bhutan OR Bolivia OR Bosnia OR Herzegovina OR Hercegovina OR Botswana OR Brasil OR Brazil OR Bulgaria OR “Burkina Faso” OR “Burkina Fasso” OR “Upper Volta” OR Burundi OR Urundi OR Cambodia OR “Khmer Republic” OR Kampuchea OR Cameroon OR Cameroons OR Cameron OR Cape Verde OR “Central African Republic” OR Chad OR China OR Colombia OR Comoros OR Comoro Islands OR Comores OR Mayotte OR Congo OR Zaire OR Costa Rica OR “Cote d'Ivoire” OR “Ivory Coast” OR Cuba OR Djibouti OR “French Somaliland” OR Dominica OR “Dominican Republic” OR “East Timor” OR “East Timur” OR “Timor Leste” OR Ecuador OR Egypt OR “El Salvador” OR Eritrea OR Ethiopia OR Fiji OR Gabon OR “Gabonese Republic” OR Gambia OR Gaza OR Georgia OR Ghana OR Gold Coast OR Grenada OR Guatemala OR Guinea OR Guam OR Guiana OR Guyana OR Haiti OR Honduras OR India OR Indonesia OR Iran OR Iraq OR Jamaica OR Jordan OR Kazakhstan OR Kenya OR Kiribati OR Kosovo OR Kyrgyzstan OR Kirghizia OR “Kyrgyz Republic” OR Kirghiz OR Kirgizstan OR “Lao PDR” OR Laos OR Latvia OR Lebanon OR Lesotho OR Liberia OR Libya OR Macedonia OR Madagascar OR Malaysia OR Malaya OR Malay OR Maldives OR Malawi OR Mali OR Marshall Islands OR Mauritania OR Mauritius OR Mexico OR Micronesia OR Moldova OR Moldovia OR Mongolia OR Montenegro OR Morocco OR Mozambique OR Myanmar OR Myanma OR Burma OR Namibia OR Nepal OR Nicaragua OR Niger OR Nigeria OR Pakistan OR Panama OR Paraguay OR Peru OR Philippines OR Philipines OR Phillipines OR Romania OR Rumania OR Roumania OR Russia OR Russian OR Rwanda OR Ruanda OR “Saint Lucia” OR “St Lucia” OR “Saint Vincent” OR “St Vincent” OR Grenadines OR Samoa OR Sao Tome OR Senegal OR Serbia OR Montenegro OR “Sierra Leone” OR “Sri Lanka” OR Ceylon OR “Solomon Islands” OR Somalia OR Sudan OR Suriname OR Surinam OR Swaziland OR Eswatini OR “South Africa” OR Syria OR Tajikistan OR Tadzhikistan OR Tadjikistan OR Tanzania OR Thailand OR Togo OR Tonga OR Tunisia OR Turkey OR Turkmenistan OR Tuvalu OR Uganda OR Ukraine OR Russia OR USSR OR “Soviet Union” OR Uzbekistan OR Uzbek OR Vanuatu OR Venezuela OR Vietnam OR “Viet Nam” OR “West Bank” OR Yemen OR Zambia OR Zimbabwe OR “Sub Saharan Africa” OR “SubSaharan Africa”)
**Combined search syntax**						
(hypertens* OR “high blood pressure” OR “blood pressure” OR diabet* OR dm2 OR niddm OR dm 2 OR t2d OR “dm type 2” OR “dm type II” OR dm1 OR iddm OR “dm 1” OR t1d OR “dm type 1” OR “dm type I” OR "Diabetes Mellitus") AND (“support group*” OR buddy OR “self-help group*” OR “peer group*” OR “informal group*” OR “social group” OR volunteer* OR “Self-help groups" OR "peer microfinance" OR "group medical visits") AND (financ* OR cash OR fund OR contribution OR support OR backing OR aid OR microfinance)						
