# Supplementary material for: Effectiveness of self‐financing patient‐led support groups in the management of hypertension and diabetes in low‐ and middle‐income countries: Systematic review
Source: Trop Med Int Health. 2022 Dec 23;28(2):80–9. doi: 10.1111/tmi.13842 (PMC10107175; doi:10.1111/tmi.13842)
Supplement: Supplementary file 1 — Data S1: Supporting Information [file TMI-28-80-s001.zip › 6. Online supplemental file 3_Data extraction form.pdf]

Systematic review data extraction form

REFERENCE AND OBJECTIVE			POPULATION					INTERVENTION/ COMPARATOR (WHERE APPLICABLE)		OUTCOME	STUDY QUALITY AND RISK OF BIAS		COMMENTS	
Authors, title, journal	Year published	Purpose/objective	Study method/ design	Subject characteristics	Setting/country	Year data collected	Sample size	Intervention	Control	Key results/ findings	Risk of bias assessment	Study quality assessment	Comments on methodology, limitations, generalisability	Other comments
