# Supplementary material for: Effectiveness of self‐financing patient‐led support groups in the management of hypertension and diabetes in low‐ and middle‐income countries: Systematic review
Source: Trop Med Int Health. 2022 Dec 23;28(2):80–9. doi: 10.1111/tmi.13842 (PMC10107175; doi:10.1111/tmi.13842)
Supplement: Supplementary file 1 — Data S1: Supporting Information [file TMI-28-80-s001.zip › 7. Online supplemental file 4_Study quality and risk of bias.pdf]

Supplementary table 2. Study quality assessment: National Institute of Health quality assessment for randomised controlled trials (Vedanthan et al., 2021)

Questions	Yes	No	Other (CD,NR,NA)*
1. Was the study described as randomized, a randomized trial, a randomized clinical trial, or an RCT	√		
2. Was the method of randomization adequate (i.e., use of randomly generated assignment)?	√		
3. Was the treatment allocation concealed (so that assignments could not be predicted)?	√		
4. Were study participants and providers blinded to treatment group assignment?		√	
5. Were the people assessing the outcomes blinded to the participants' group assignments?		√	
6. Were the groups similar at baseline on important characteristics that could affect outcomes (e.g., demographics, risk factors, co-morbid conditions)?	√		
7. Was the overall drop-out rate from the study at endpoint 20% or lower of the number allocated to treatment?	√		
8. Was the differential drop-out rate (between treatment groups) at endpoint 15 percentage points or lower?	√		
9. Was there high adherence to the intervention protocols for each treatment group?	√		
10. Were other interventions avoided or similar in the groups (e.g., similar background treatments)?	√		
11. Were outcomes assessed using valid and reliable measures, implemented consistently across all study participants?	√		
12. Did the authors report that the sample size was sufficiently large to be able to detect a difference in the main outcome between groups with at least 80% power?	√		
13. Were outcomes reported or subgroups analysed pre-specified (i.e., identified before analyses were conducted)?	√		
14. Were all randomized participants analysed in the group to which they were originally assigned, i.e., did they use an intention-to-treat analysis?	√		
**Rating** (Good, Fair, Poor)	**Fair**		

*CD, cannot determine; NA, not applicable; NR, not reported**Supplementary table 3. Study quality assessment: National Institute of Health quality assessment for cohort Studies (Taniguchi et al., 2017)**

**Questions**	**Yes**	**No**	**Other (CD,NR,NA)***
1. Was the research question or objective in this paper clearly stated?	√		
2. Was the study population clearly specified and defined?	√		
3. Was the participation rate of eligible persons at least 50%?	√		
4. Were all the subjects selected or recruited from the same or similar populations (including the same time period)? Were inclusion and exclusion criteria for being in the study prespecified and applied uniformly to all participants?	√		
5. Was a sample size justification, power description, or variance and effect estimates provided?		√	
6. For the analyses in this paper, were the exposure(s) of interest measured prior to the outcome(s) being measured?	√		
7. Was the timeframe sufficient so that one could reasonably expect to see an association between exposure and outcome if it existed?	√		
8. For exposures that can vary in amount or level, did the study examine different levels of the exposure as related to the outcome (e.g., categories of exposure, or exposure measured as continuous variable)?			√ (N/A)
9. Were the exposure measures (independent variables) clearly defined, valid, reliable, and implemented consistently across all study participants?	√		
10. Was the exposure(s) assessed more than once over time?			V (N/A)
11. Were the outcome measures (dependent variables) clearly defined, valid, reliable, and implemented consistently across all study participants?	√		
12. Were the outcome assessors blinded to the exposure status of participants?			√ (NR)
13. Was loss to follow-up after baseline 20% or less?	√		
14. Were key potential confounding variables measured and adjusted statistically for their impact on the relationship between exposure(s) and outcome(s)?	√		
**Rating** (Good, Fair, Poor)	Fair		

*CD, cannot determine; NA, not applicable; NR, not reported

Supplementary table 4. Study quality assessment: National Institute of Health quality assessment for before and after studies (Pastakia et al., 2017)

Questions	Yes	No	Other (CD,NR,NA)*
1. Was the study question or objective clearly stated?	√		
2. Were eligibility/selection criteria for the study population prespecified and clearly described?	√		
3. Were the participants in the study representative of those who would be eligible for the test/service/intervention in the general or clinical population of interest?	√		
4. Were all eligible participants that met the prespecified entry criteria enrolled?	√		
5. Was the sample size sufficiently large to provide confidence in the findings?			√ (NR))
6. Was the test/service/intervention clearly described and delivered consistently across the study population?	√		
7. Were the outcome measures prespecified, clearly defined, valid, reliable, and assessed consistently across all study participants?	√		
8. Were the people assessing the outcomes blinded to the participants' exposures/interventions?			√ (NR)
9. Was the loss to follow-up after baseline 20% or less? Were those lost to follow-up accounted for in the analysis?	√		
10. Did the statistical methods examine changes in outcome measures from before to after the intervention? Were statistical tests done that provided p values for the pre-to-post changes?	√		
11. Were outcome measures of interest taken multiple times before the intervention and multiple times after the intervention (i.e., did they use an interrupted time-series design)?	√		
12. If the intervention was conducted at a group level (e.g., a whole hospital, a community, etc.) did the statistical analysis take into account the use of individual-level data to determine effects at the group level?	√		
**Rating** (Good, Fair, Poor)	Fair		

*CD, cannot determine; NA, not applicable; NR, not reported

Supplementary table 5. Risk of bias assessment using the Revised Cochrane risk-of-bias tool for cluster-randomized trials (RoB 2 CRT) version 18 March 2021

Study details

**Reference**	Vedanthan, R., Kamano, J.H., Chrysanthopoulou, S.A., Mugo, R., Andama, B., Bloomfield, G.S., Chesoli, C.W., DeLong, A.K., Edelman, D., Finkelstein, E.A. and Horowitz, C.R.

Study design

☐ Individually randomised parallel-group trial
X Cluster-randomized parallel-group trial
☐ Individually randomized cross-over (or other matched) trial

For the purposes of this assessment, the interventions being compared are defined as

Experimental:	**x**	Comparator:	

**Specify which outcome is being assessed for risk of bias**	The impact of microfinance and group medical visits on systolic blood pressure and cardiovascular risk reduction
**Specify the numerical result being assessed.**	A decrease in systolic blood pressure was recorded in all 4 intervention arms (Usual care [UC]= -11.4, Microfinance[MF] onlY 14.8, Group medical visits [GMV] 14.7, and GMV and MF 16.4). Modelling shows that compared to the UC arm reduction in the MF-GMV arm was 3.9 mm Hg more (p=0.09). Blood pressure control at 12 months was achieved in 40.2% of participants. To benefit more than older individuals. without health insurance and lower wealth or employment experienced greater SBP reductions in the MF and GMV-MF arms. 5, 4, 13, and 13 deaths in the UC, MF, GMV, and GMV-MF arms, respectively. No systematic difference in mortality rates.

Is the review team's aim for this result…?

✕ to assess the effect of *assignment to intervention* (the 'intention-to-treat' effect)

✕ to assess the effect of *adhering to intervention* (the 'per-protocol' effect)

If the aim is to assess the effect of *adhering to intervention*, select the deviations from the intended intervention that should be addressed (at least one must be checked):

☐ occurrence of non-protocol interventions
✕ failures in implementing the intervention that could have affected the outcome
✕ non-adherence to their assigned intervention by trial participants

Which of the following sources were obtained to help inform the risk-of-bias assessment? (tick as many as apply)

✕ Journal article(s) with results of the trial
✕ Trial protocol
✕ Statistical analysis plan (SAP)
✕ Non-commercial trial registry record (e.g. ClinicalTrials.gov record)
☐ Company-owned trial registry record (e.g. GSK Clinical Study Register record)
✕ "Grey literature" (e.g. unpublished thesis)
✕ Conference abstract(s) about the trial
☐ Regulatory document (e.g. Clinical Study Report, Drug Approval Package)
✕ Research ethics application
☐ Grant database summary (e.g. NIH RePORTER or Research Councils UK Gateway to Research)
✕ Personal communication with trialist
✕ Personal communication with the sponsor

Risk of bias assessment

Responses underlined in green are potential markers for low risk of bias, and responses in red are potential markers for risk of bias. Where questions relate only to signposts to other questions, no formatting is used.

Domain 1a: Risk of bias arising from the randomization process

**Signalling questions**	**Comments**	**Response options**
**1a.1 Was the allocation sequence random?**	Blinded biostatistician randomised via module 'ralloc' of the statistical software Stata (StataCorp, College Station, TX), stratified by clinic location in the state of Sonora (North, Central, South), with permuted blocks to maintain the balance between arms.	**Y**
**1a.2 Was the allocation sequence concealed until clusters were enrolled and assigned to interventions?**		**N**

	**<u>The participants and research staff could not practicably be blinded to intervention assignments.</u>**	
**1a.3 Did baseline differences between intervention groups suggest a problem with the randomization process?**	The differences are compatible with chance or likely to be because of identification, recruitment procedures and participant characteristics	**N**
**Risk-of-bias judgement**	Potential for identification/recruitment bias because participants who know about cluster allocation before being recruited (in all 4 intervention arms)	**Some concerns**
Optional: What is the predicted direction of bias arising from the randomization process?		**Favours experimental**
**Domain 1b: Risk of bias arising from the timing of identification or recruitment of participants in a cluster-randomized trial**		
**Signalling questions**	**Comments**	**Response options**
**1b.1 Were all the individual participants identified and recruited (if appropriate) before the randomization of clusters?**	All participants were identified and recruited before the clusters were randomized	**<u>Y</u>**
**1b.2 <u>If N/PN/NI to 1b.1</u>: Is it likely that the selection of individual participants was affected by knowledge of the intervention assigned to the cluster?**	Recruiting individuals were aware of cluster allocation before recruitment	**Y**
**1b.3 Were there baseline imbalances that suggest differential identification or recruitment of individual participants between intervention groups?**	There were imbalances due to the numbers of participants recruited into each group and variations in their characteristics. Such imbalances are more common in cluster-randomized trials than imbalances due to problems with randomization.	**Y**
**Risk-of-bias judgement**	Those identifying potential participants (when recruitment is to take place subsequently) are aware of cluster allocation and are likely, consciously or subconsciously, to have differentially included potential individual participants in different intervention groups	**High**
Optional: What is the predicted direction of bias arising from the timing of identification and recruitment of participants?	Recruiting individuals were aware of cluster allocation before recruitment	**Favours experimental**
**Domain 2: Risk of bias due to deviations from the intended interventions (*effect of assignment to intervention*)**		
**Signalling questions**	**Comments**	**Response options**

**2.1a Were participants aware that they were in a trial?**	Participants were aware that they were in a trial.	Y
**2.1b. If Y/PY/NI to 2.1a: Were participants aware of their assigned intervention during the trial?**	Participants were aware of the assigned intervention during the trial	Y
**2.2. Were carers and people delivering the interventions aware of participants' assigned intervention during the trial?**	Investigators were involved in caring for participants and were aware of the assigned intervention.	Y
**2.3. If Y/PY/NI to 2.1 or 2.2: Were there deviations from the intended intervention that arose because of the trial context?**	Deviations from the intended intervention that arise due to the trial context are rarely reported in cluster-randomized trials and may occur rarely. However, there is no information available in this study	NI
**2.4 If Y/PY to 2.3: Were these deviations likely to have affected the outcome?**	There is no information available in this study	NI
**2.5. If Y/PY/NI to 2.4: Were these deviations from the intended intervention balanced between groups?**	There is no information available in this study	NI
**2.6 Was an appropriate analysis used to estimate the effect of assignment to intervention?**	Data for each participant were collected at baseline and 3 and 12 months.<br>mean SD for continuous measurements, and frequencies and percentages for categorical were reported. using fitted linear mixed-effects models to estimate the effect of the intervention on primary and secondary outcomes, using random effects to account for clustering of individuals within the health facility catchment area.	Y
**2.7 If N/PN/NI to 2.6: Was there potential for a substantial impact (on the result) of the failure to analyse participants in the group to which they were randomized?**	There is the possibility of cross-contamination across the trial arms. However, this the scenario is unlikely since the clusters were geographically distinct areas and participants were invited by the research staff to attend the group sessions;<br>in addition, no participants in the UC arm reported having attended a GMV.	N
**Risk-of-bias judgement**	Participants were aware of the assigned intervention during the trial	**Some concerns**
Optional: What is the predicted direction of bias due to deviations from intended interventions?	At the individual participant's level (e.g. a self-management course)<br>At the health professionals' level (e.g. feedback on some aspects of care, educational sessions)	**Favours experimental**
**Domain 2: Risk of bias due to deviations from the intended interventions (*effect of adhering to intervention*)**		
**Signalling questions**	**Comments**	**Response options**

**2.1. Were participants aware of their assigned intervention during the trial?**	Participants were aware of the assigned intervention during the trial	**Y**
**2.2. Were carers and people delivering the interventions aware of participants' assigned intervention during the trial?**	Investigators involved in caring for participants are aware of the assigned intervention, then implementation of the intended intervention	**Y**
**2.3. [If applicable:] If Y/PY/NI to 2.1 or 2.2: Were important non-protocol interventions balanced across intervention groups?**	No information reported	NI
**2.4. [If applicable:] Were there failures in implementing the intervention that could have affected the outcome?**	The per-protocol analysis yielded estimates of SBP reductions relative to UC that were greater in magnitude than the intention to treat analysis, particularly in the GMV-MF and GMV arms (Table 2). On average during the 12-month follow-up period, the median numbers (and percentages) of group sessions attended by participants in the different intervention arms were 9 (75%), 7 (67%), and 10 (83%), for the MF, GMV, and GMV-MF arms, respectively. Median attendance at each group session ranged from 10 to 16 individuals across all intervention arms.	N
**2.5. [If applicable:] Was there non-adherence to the assigned intervention regimen that could have affected participants' outcomes?**	There is no information available in this study	**NI**
**2.6. If N/PN/NI to 2.3, or Y/PY/NI to 2.4 or 2.5: Was an appropriate analysis used to estimate the effect of adhering to the intervention?**	Data for each participant were collected at baseline and 3 and 12 months. mean SD for continuous measurements, and frequencies and percentages for categorical were reported. using fitted linear mixed-effects models to estimate the effect of the intervention on primary and secondary outcomes, using random effects to account for clustering of individuals within the health facility catchment area.	**Y**
**Risk-of-bias judgement**	The per-protocol analysis yielded estimates of SBP reductions relative to UC that were greater in magnitude than the intention to treat analysis, particularly in the GMV-MF and GMV arms (Table 2).	**Low risk**
Optional: What is the predicted direction of bias due to deviations from intended interventions?	At the individual participant's level (e.g. a self-management course)<br>At the health professionals' level (e.g. feedback on some aspects of care, educational sessions)	**Favours experimental**
**Domain 3: Risk of bias due to missing outcome data**		
**Signalling questions**	**Comments**	**Response options**

**3.1a Were data for this outcome available for all clusters that recruited participants?**	A per-protocol analysis using randomization as an instrumental variable to assess the effect of full participation in each intervention, relative to UC, on SBP change. Participation was defined as the percentage of group sessions (either GMV, MF, or GMV-MF) attended by the participant among those organized by his/her group during the study follow-up period.	**Y**
**3.1b Were data for this outcome available for all, or nearly all, participants within clusters?**	A total of 2,890 individuals (69.9% women) were enrolled (708 UC, 709 MF, 740 GMV, and 733 GMV-MF). The average baseline SBP was 157.5 mm Hg. Mean SBP declined 11.4, 14.8, 14.7, and 16.4 mm Hg in UC, MF, GMV, and GMV-MF, respectively. Adjusted estimates and multiplicity-adjusted 98.3% confidence intervals showed that, relative to UC, SBP reduction was 3.9 mm Hg (8.5 to 0.7), 3.3 mm Hg (7.8 to 1.2), and 2.3 mm Hg (7.0 to 2.4) greater in GMVMF, GMV, and MF, respectively. GMV and GMV-MF tended to benefit women, and MF and GMV-MF tended to benefit poorer individuals. Active participation in GMV-MF was associated with greater benefit	**Y**
**3.2 <u>If N/PN/NI to 3.1a or 3.1b</u>: Is there evidence that the result was not biased by missing data?**	the clusters were geographically distinct areas and participants were invited by the research staff to attend the group sessions; in addition, no participants in the UC arm reported having attended a GMV.	**Y**
**3.3 <u>If N/PN to 3.2</u> Could missingness in the outcome depend on its true value?**	There is no information available in this study	NI
**3.4 <u>If Y/PY/NI to 3.3</u>: Is it likely that missingness in the outcome depended on its true value?**	There is no information available in this study	NI
**Risk-of-bias judgement**	A per-protocol analysis using randomization as an instrumental variable to assess the effect of full participation in each intervention	**Low risk**
Optional: What is the predicted direction of bias due to missing outcome data?	Participation was defined as the percentage of group sessions (either GMV, MF, or GMV-MF) attended by the participant among those organized by his/her group during the study follow-up period.	**Favours experimental**
**Domain 4: Risk of bias in the measurement of the outcome**		
**Signalling questions**	**Comments**	**Response options**
**4.1 Was the method of measuring the outcome inappropriate?**	The primary outcome measure was 1-year absolute mean change in SBP, measured by trained study staff using electronic BP machines and standardized procedures. Key secondary outcome measures included change in DBP and change in 10-year CVD event risk as measured by the QResearch-based QRISK3 score.	N

	Other secondary outcomes included BP control (SBP <140 mm Hg and DBP <90 mm Hg), change in total and low-density lipoprotein cholesterol, change in the International Wealth Index (asset-based index of a household's material well-being, used in LMICs). and change in livestock ownership.<br>Pre-specified subgroup analysis included assessing the change in the primary outcome (i.e., SBP) in the following groups: sex, age, baseline health insurance status, baseline monthly earnings, baseline wealth category, baseline livestock ownership, baseline land ownership, and recruitment pathway.	
**4.2 Could measurement or ascertainment of the outcome have differed between intervention groups?**	In the GMV-MF arm, the monthly GMV was integrated into the MF groups, wherein each group meeting consisted of an initial MF portion, followed by the GMV. Thus, participants received their clinical care in a GMV as well as participated in MF	N
**4.3a If N/PN/NI to 4.1 and 4.2: Were outcome assessors aware that a trial was taking place?**	Investigators were aware of the assigned intervention and implementation of the intended intervention.	**Y**
**4.3b If Y/PY/NI to 4.3a: Were outcome assessors aware of the intervention received by study participants?**	Investigators were involved in caring for participants and were aware of the assigned intervention.	**Y**
**4.4 If Y/PY/NI to 4.3b: Could assessment of the outcome have been influenced by knowledge of intervention received?**	There is the possibility of cross-contamination across the trial arms. However, this scenario is unlikely since the clusters were geographically distinct areas and participants were invited by the research staff to attend the group sessions; in addition, no participants in the UC arm reported having attended a GMV.	**PY**
**4.5 If Y/PY/NI to 4.4: Is it likely that assessment of the outcome was influenced by knowledge of intervention received?**		**PY**
**Risk-of-bias judgement**	Investigators are involved in caring for participants and are aware of the assigned intervention.	**High Risk**
Optional: What is the predicted direction of bias in the measurement of the outcome?	In the GMV-MF arm, the monthly GMV was integrated into the MF groups, wherein each group meeting consisted of an initial MF portion, followed by the GMV. Thus, participants received their clinical care in a GMV as well as participated in MF.	**Favours experimental**
**Domain 5: Risk of bias in the selection of the reported result**		
**Signalling questions**	**Comments**	**Response options**

**5.1 Were the data that produced this result analysed in accordance with a pre-specified analysis plan that was finalized before unblinded outcome data were available for analysis?**	The per-protocol analysis produced estimates of SBP reductions relative to UC that were greater in magnitude than the intention to- treat analysis,	Y
**Is the numerical result being assessed likely to have been selected, based on the results, from...**		
**5.2. ... multiple eligible outcome measurements (e.g. scales, definitions, time points) within the outcome domain?**	The primary outcome measure was a one-year absolute mean change in SBP, measured by trained study staff using electronic BP machines and standardized procedures<br>Secondary outcome measures included change in DBP and change in 10-year CVD event risk as measured by the Q Research-based QRISK3 score	**Y**
**5.3 ... multiple eligible analyses of the data?**	For each participant were collected at baseline and 3 and 12 months. mean SD for continuous measurements and frequencies and percentages for categorical variables were reported. Using fitted linear mixed-effects models to estimate the effect of the intervention on primary and secondary outcomes, using random effects to account for clustering of individuals within the health facility catchment area	**Y**
**Risk-of-bias judgement**	The authors conducted a cluster-randomized trial with 4 arms and 24 clusters: 1) usual care (UC); 2) usual care plus microfinance (MF); 3) group medical visits (GMVs); and 4) GMV integrated into MF (GMV-MF). The primary outcome was a one-year change in systolic blood pressure (SBP). Mixed-effects intention-to-treat models were used to evaluate the outcomes.	**Low risk**
Optional: What is the predicted direction of bias due to the selection of the reported result?	The per-protocol analysis produced estimates of SBP reductions relative to UC that were greater in magnitude than the intention to treat analysis.	**Towards null**
**Overall risk of bias**		
**Risk-of-bias judgement**	The authors conducted a cluster-randomized trial with 4 arms and 24 clusters: 1) usual care (UC); 2) usual care plus microfinance (MF); 3) group medical visits (GMVs); and 4) GMV integrated into MF (GMV-MF). The primary outcome was a one-year change in systolic blood pressure (SBP). Mixed-effects intention-to-treat models were used to evaluate the outcomes.	**Some concerns**

	A per-protocol analysis using randomization as an instrumental variable to assess the effect of full participation in each intervention, relative to UC, on SBP change. Participation was defined as the percentage of group sessions (either GMV, MF, or GMV-MF) attended by the participant among those organized by his/her group during the study follow-up period.<br>However, participants were aware of the assigned intervention during the trial and the investigators are involved in caring for participants are aware of the assigned intervention, then implementation of the intended intervention	
Optional: What is the overall predicted direction of bias for this outcome?	There is the possibility of cross-contamination across the trial arms. However, this scenario is unlikely since the clusters were geographically distinct areas and participants were invited by the research staff to attend the group sessions.<br>In addition, no participants in the UC arm reported having attended a GMV	**Favours experimental**
